# Supplementary material for: Neural and computational mechanisms of effort under the pressure of a deadline
Source: Nat Commun. 2026 Jul 28;17:9169. doi: 10.1038/s41467-026-75987-6 (PMC13522516; doi:10.1038/s41467-026-75987-6)
Supplement: Supplementary file 1 [file 41467_2026_75987_MOESM1_ESM.pdf]

Neural and Computational mechanisms of effort under the pressure of a deadline

M. Andrea Pisauro[1,2,3,4] Daniele Pollicino[3,5] , Lucy Fisher[1], Matthew A. J. Apps[1,2,3]

Supplementary Information

[1] Centre for Human Brain Health, School of Psychology, University of Birmingham, Birmingham, UK
[2] Institute for Mental Health, School of Psychology, University of Birmingham, Birmingham, UK
[3] Department of Experimental Psychology, University of Oxford, Oxford, UK
[4] School of Psychology, University of Plymouth, Plymouth, UK
[5] Department of Psychological and Behavioural Science, London School of Economics and Political Science, London, UK;

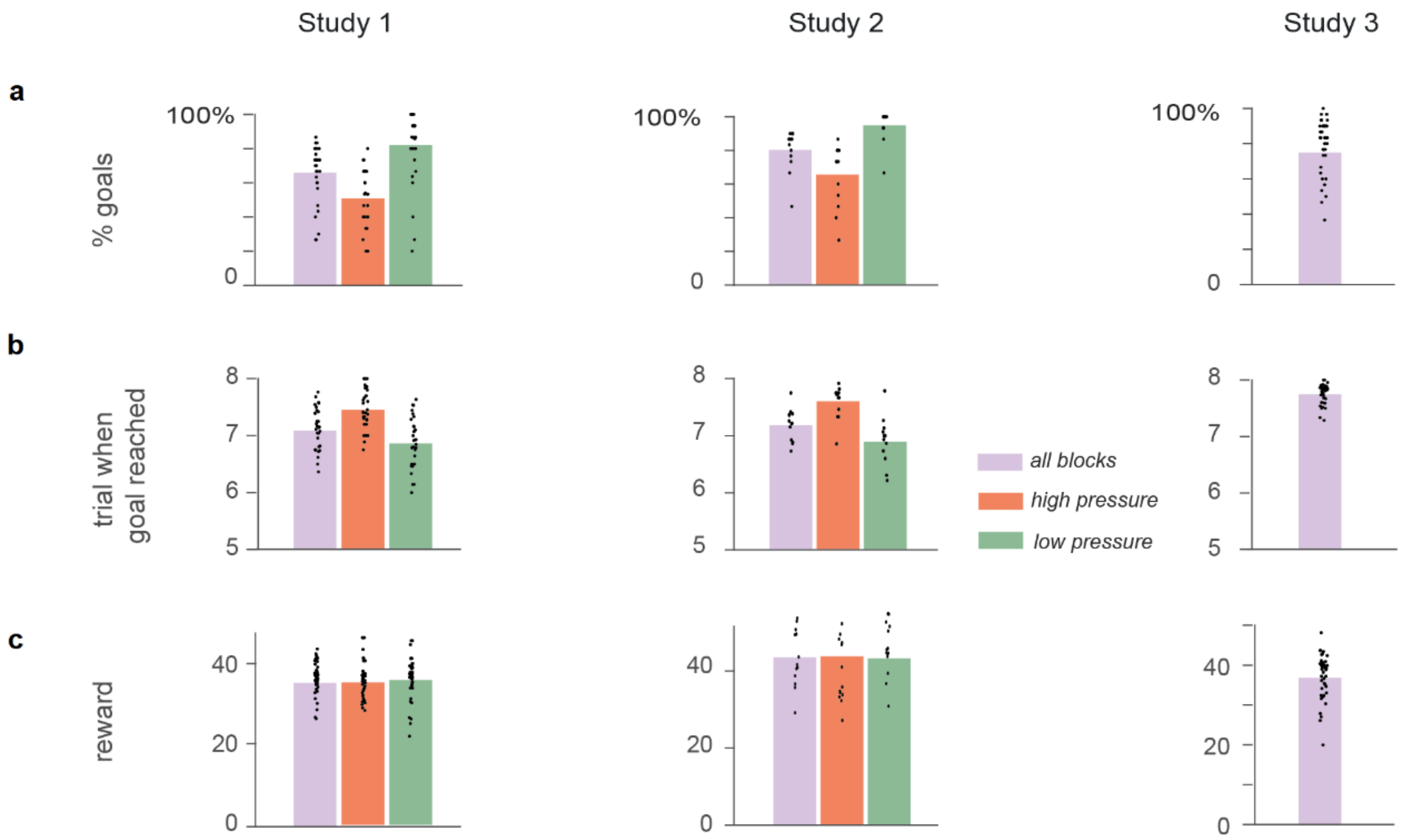

Supplementary Figure 1. *Performance in the task in Study 1 (N=30), Study 2 (N=13) and study 3 (N=38).* ***a****. Average percentage of goal reached across all, low high-pressure and low-pressure blocks in Study 1 (left), Study 2 (centre) and Study 3 (right).* ***b****. Average number of trials before deadline in which the goal was reached across all, low high-pressure and low-pressure blocks (right panel) in Study 1 (left), Study 2 (centre) and Study 3 (right).* ***c****. Average rewards collected across all, low high-pressure and low-pressure blocks (right panel) in Study 1 (left), Study 2 (centre) and Study 3 (right).*

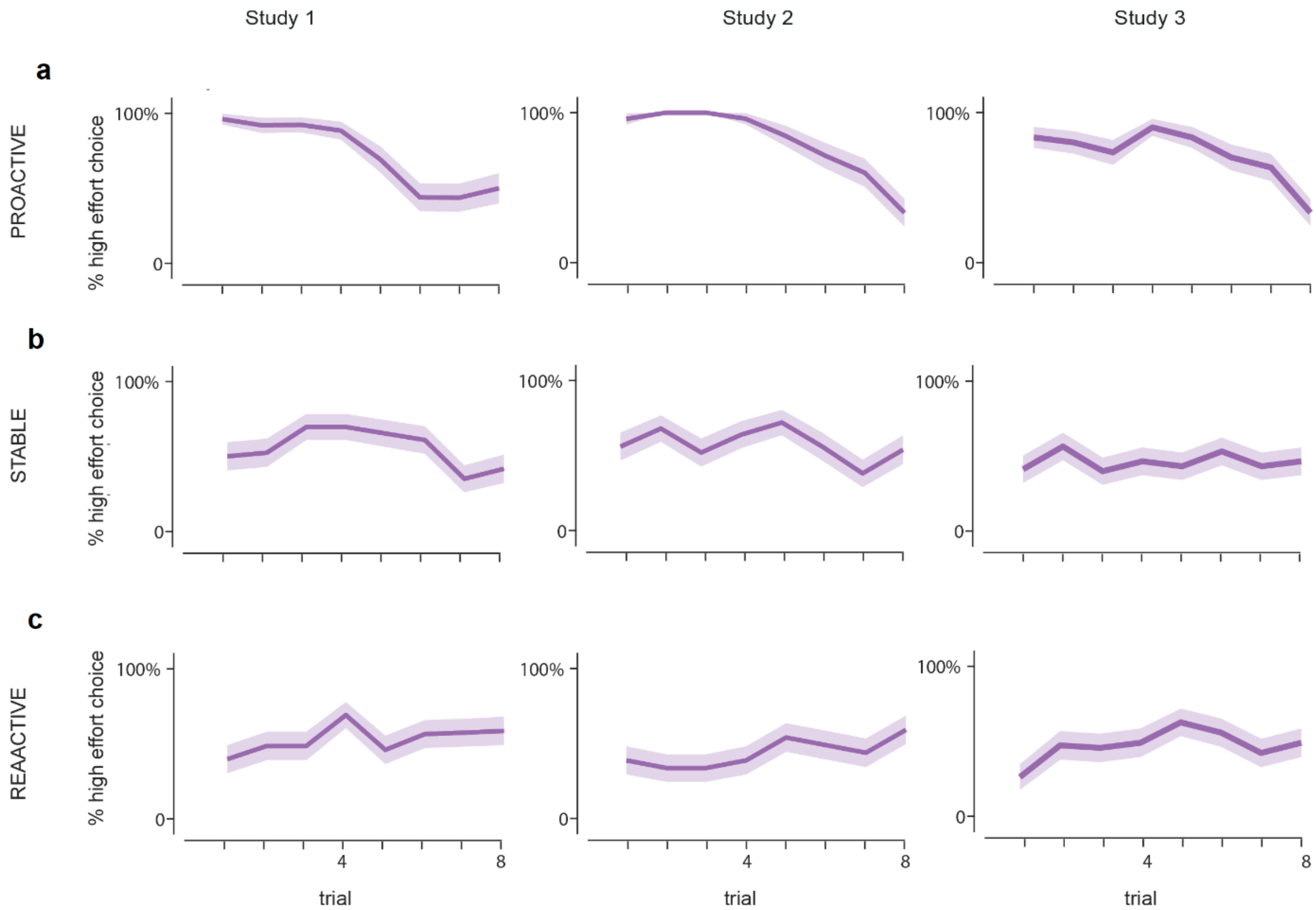

Supplementary Figure 2. *Examples of behaviour in the task.* ***a****. Individual subject average percentage of high effort choices across trials in Study 1 (left panel), Study 2 middle panel) and Study 3 (right panel). Participants could put in most effort at the beginning (proactive strategy, top row);* ***b****. distribute efforts equally across trials (stable strategy, middle row) or* ***c****. grow the likelihood to exert a high effort as time progresses (reactive strategy, bottom row).*

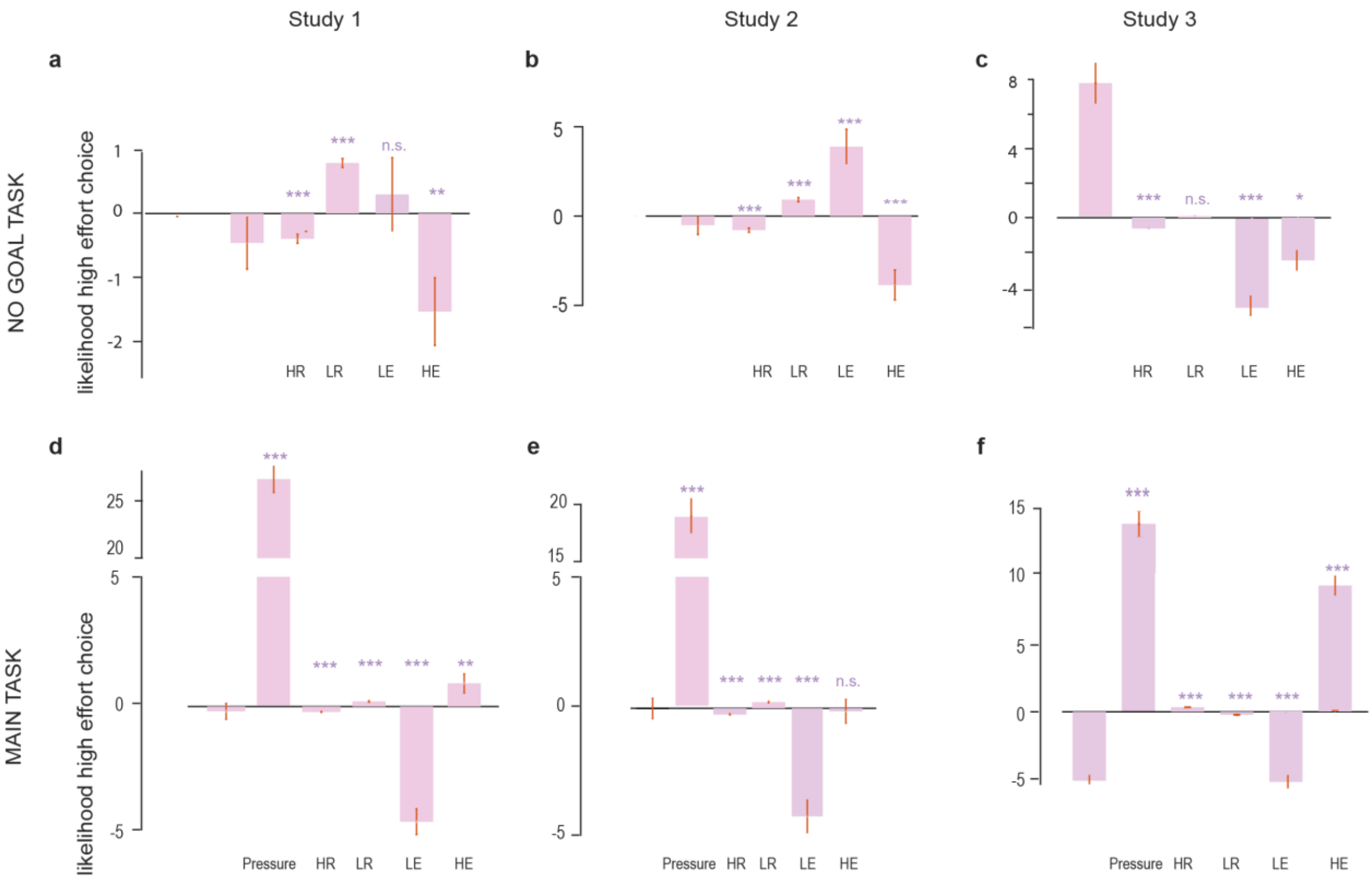

Supplementary Figure 3. *Predictors of behaviour in the task.* ***a****. A logistic regression predicting the likelihood of a High Effort Low Reward during the no-goal task in trials in Study 1,* ***b****. Same for Study 2* ***c****. Same for Study 3.* ***d****. A logistic regression predicting the likelihood of a High Effort Low Reward during the main task in Study 1.* ***e****. Same for Study 2.* ***f.*** *Same for Study 3.*

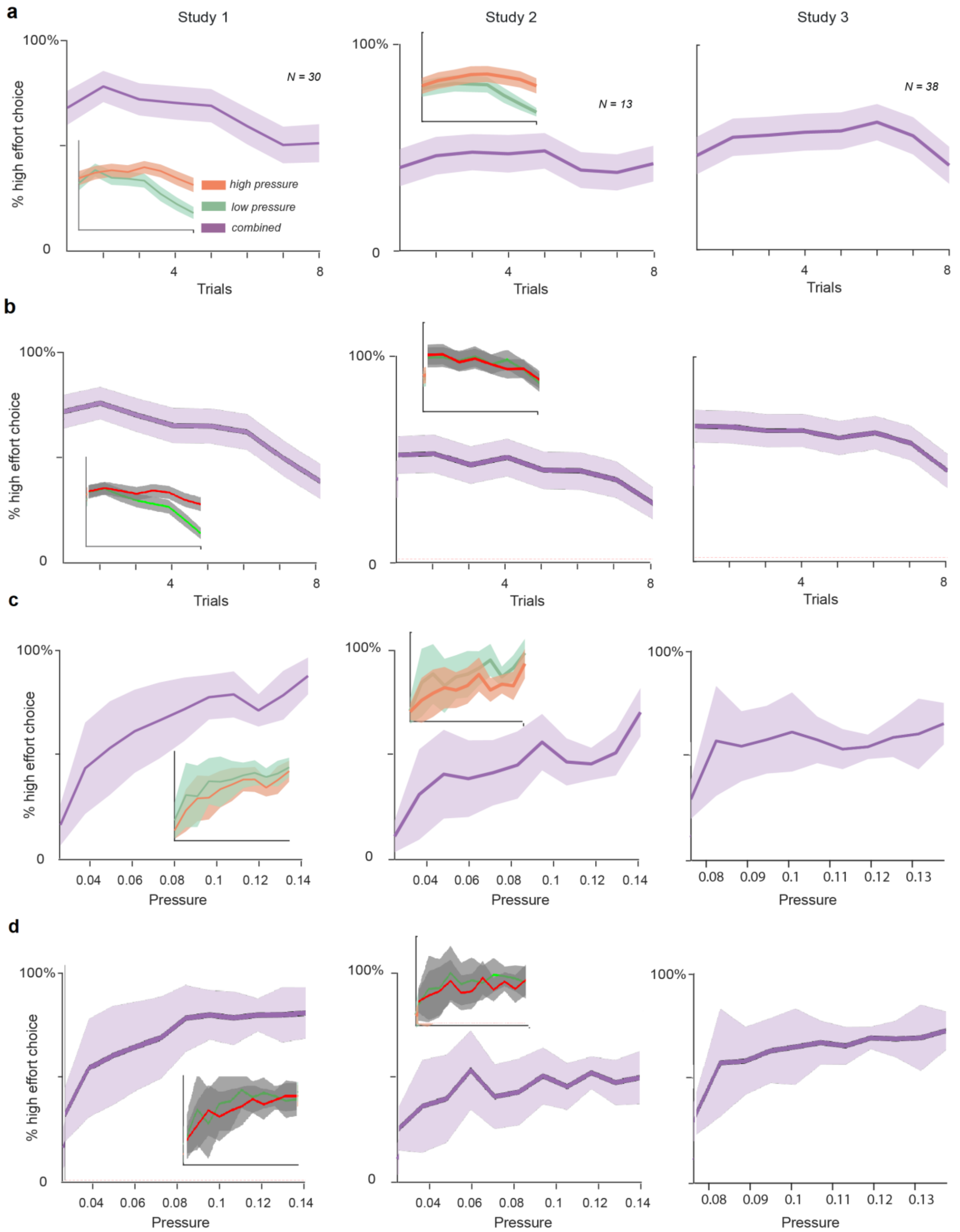

Supplementary Figure 4. *Behaviour in the task and model predictions across studies.* ***a****. Average (across subjects and blocks) percentage of high effort choices across trials for study 1. In the inset, the same for high-pressure (red) and low-pressure (green) blocks.* ***b****. Same as in* ***a.*** *for data simulated by the best model.* ***c****. Average (across subjects and blocks) percentage of high effort choices across pressure values. Pressure values where binned in 10 bins. In the inset, the same for high-pressure (red) and low-pressure (green) blocks.* ***d****. Same as in* ***c****. same for data simulated by the best model.*

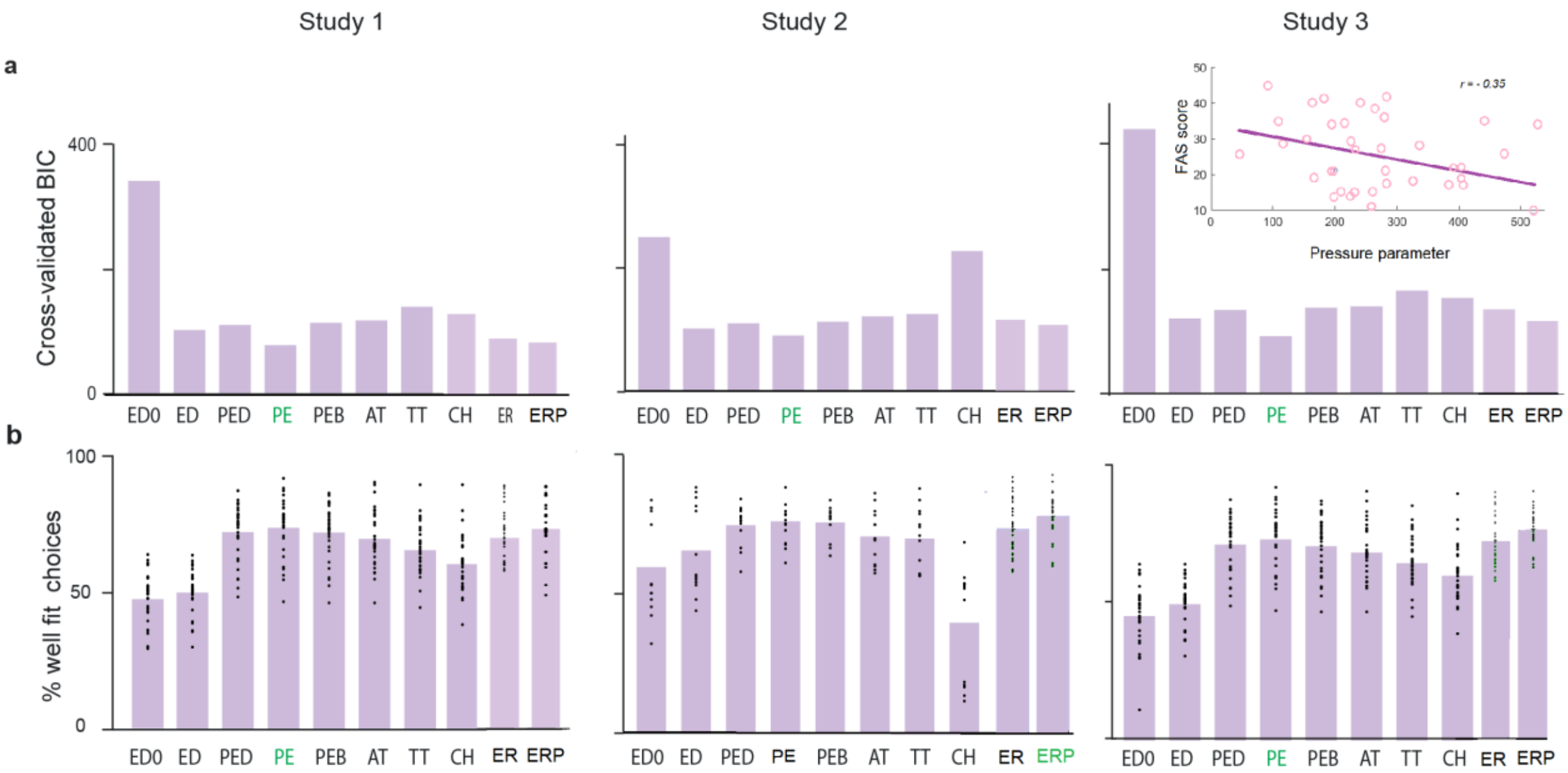

Supplementary Figure 5 *Computational modelling.* ***a****. BIC values for different computational modelling of the evaluation and choice process for Study 1 (left), Study 2 (middle) and Study 3 (right). In all cases, the winning model is PE. In study 2, ERP has a marginally higher % of well fit choices. In the inset, correlation between the pressure sensitivity parameter and the score in the Fatigue Assessment Scale, each point is a subject.* ***b*** *average percentage of correct choices across models for Study 1 (left), Study 2 (middle), Study 3 (right).*

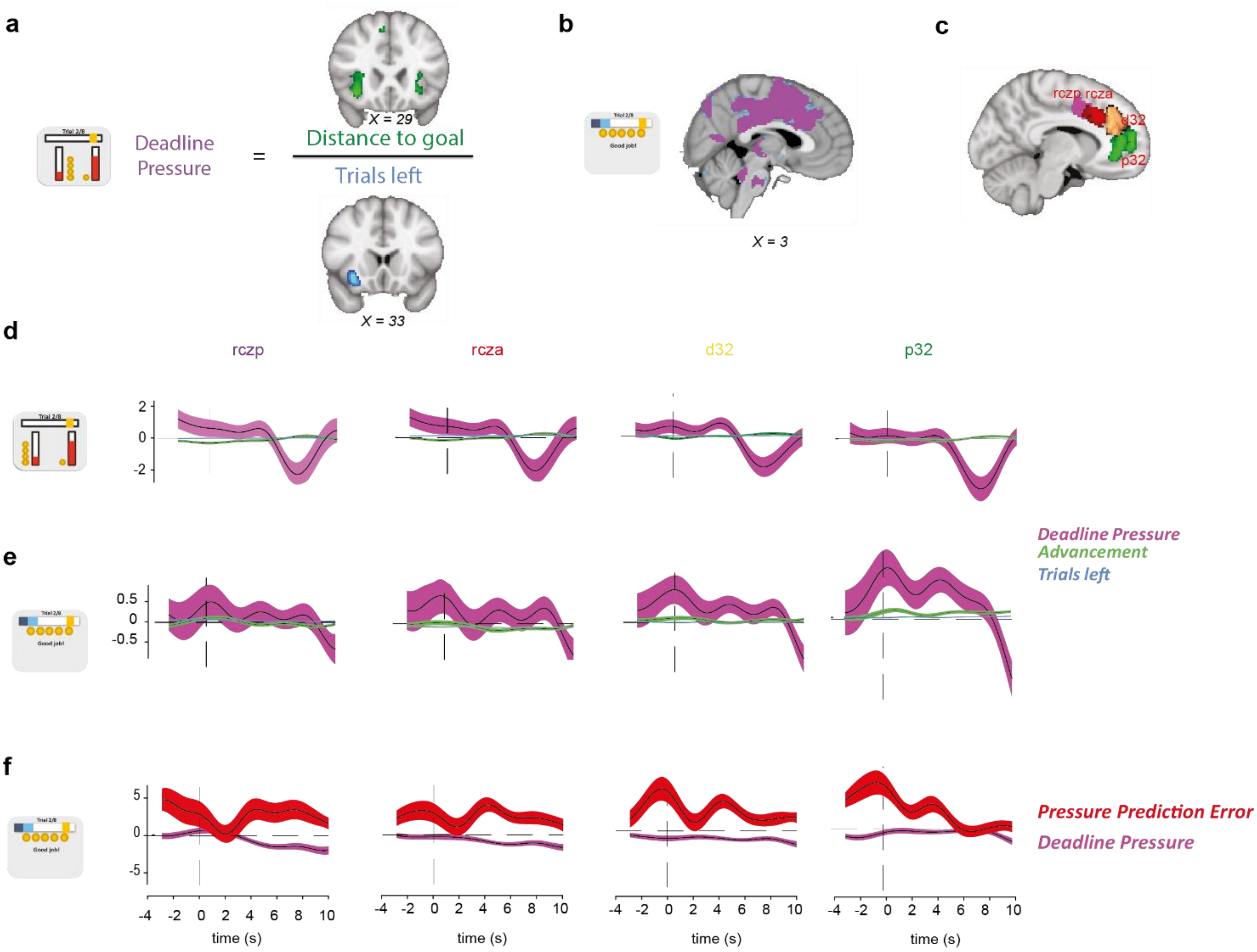

Supplementary Figure 6. *Neural representation in Middle and Anterior Cingulate Cortex.* ***a****. Representation of advancement and time left at time of offer in the right Insula.* ***b.*** *Representation of pressure at feedback time.* ***c****. Four anatomical masks for sections of the Middle and Anterior Cingulate Cortex.* ***d.*** *Time series of the strength of representation of Pressure (purple), Advancement (green), Time Left (cyan) at time of choice in voxels from the four anatomical masks.* ***e.*** *Time series of the strength of representation of Pressure (purple), Advancement (green), Time Left (cyan) at time of feedback in voxels from the four anatomical masks.* ***f****. Time series of the strength of representation of Pressure (purple) and Pressure prediction error (red) at time of feedback in voxels from the four anatomical masks.*

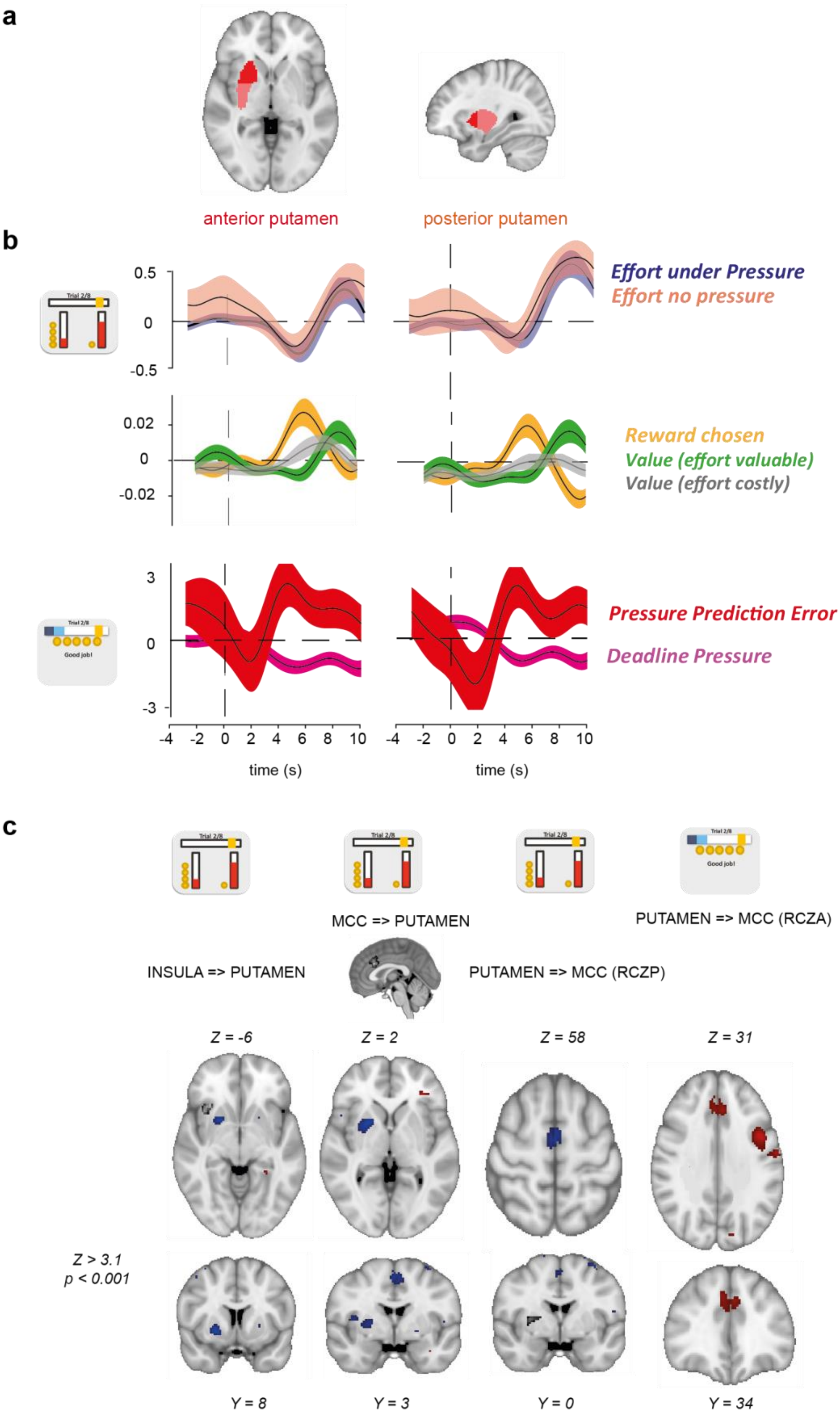

Supplementary Figure 7. *Neural representation in anterior and posterior Putamen.* ***a.*** *Anatomical subdivision of Putamen into anterior and posterior subregions.* ***b*** *Time series of the strength of representation of Effort, Reward, Value (at time of choice), Pressure and Pressure prediction error (at*

time of feedback) in voxels from the anterior and posterior Putamen anatomical ROIs. ***c****. PPI analysis using clusters in Insula (first column) and Anterior Cingulate Cortex (second column) as a seed revealed an inverse coupling with the posterior putamen. PPI analysis using clusters in Putamen at time of choice (third column) or feedback (fourth column) as a seed revealed significant coupling with regions in RCZp and RCZa respectively.*

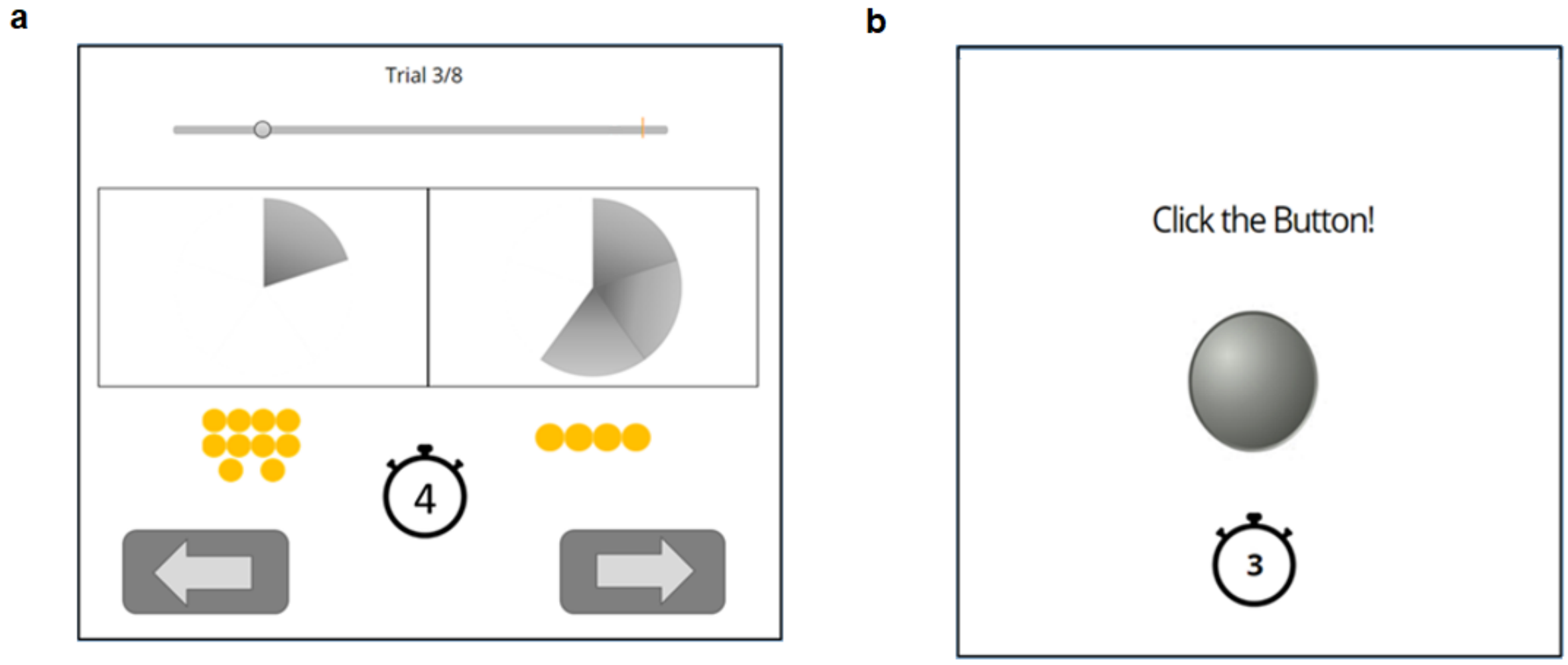

Supplementary Figure 8. *Description of the task for study 3 and 4, performed online.* ***a****. Participants need to reach a goal making enough progress over 8 trials. The goal is represented by a little yellow line on the right hand side of the bar signalling the subject progress (top). In each trial subject are offered a choice between two different levels of progress, represented by the slices in a pie chart, in exchange of two different amounts of reward, represented by the yellow coins. The choice was made by pressing either the left or right arrow buttons.* ***b****. In study 3, after each decision, subjects are asked to click for the number of times associated with the chosen effort level. In this study the goal can only be reached by clicking a certain amount of times over the 8 trials. In study 4 subjects progress without making any effort.*

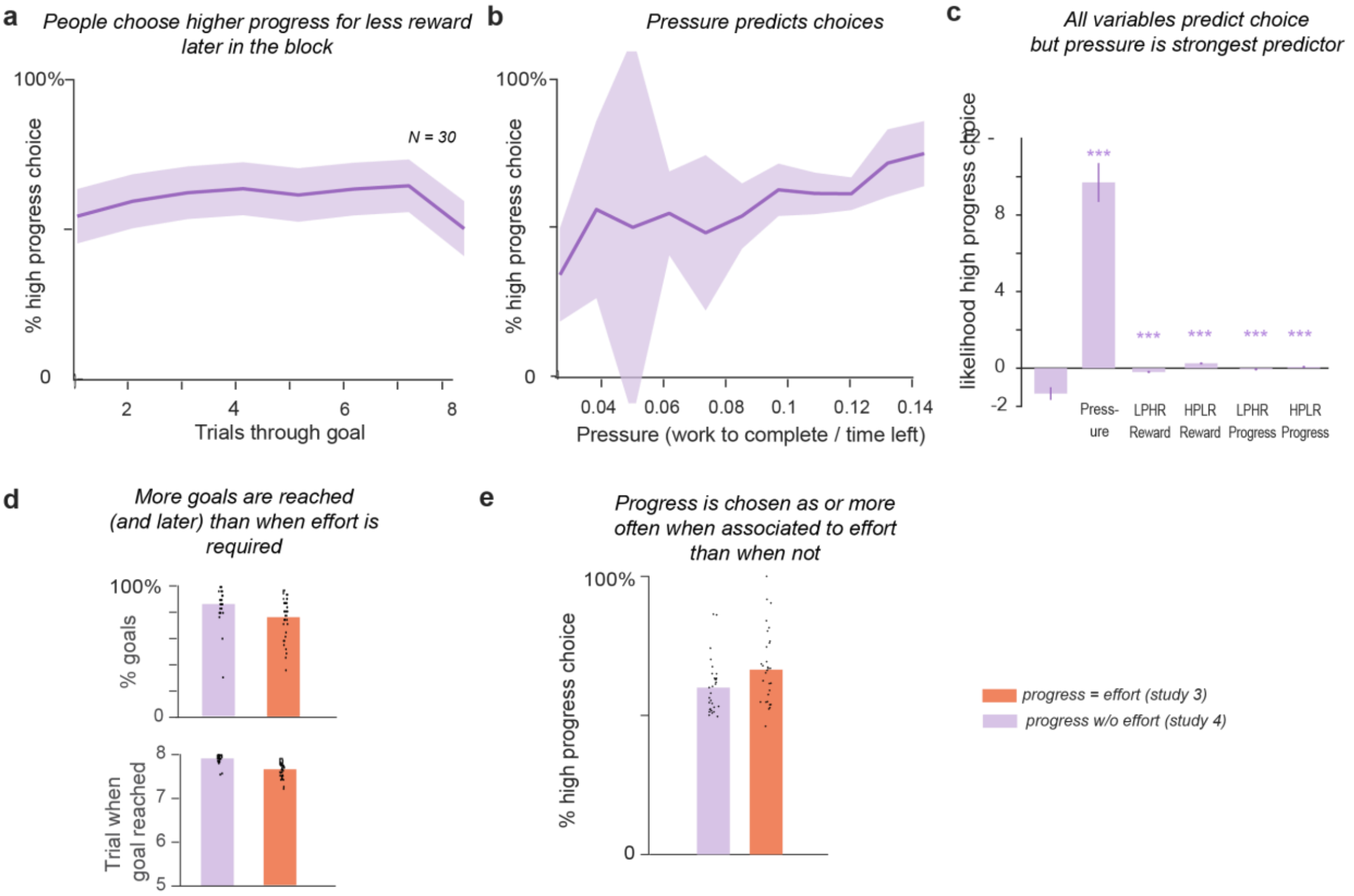

Supplementary Figure 9. *Behaviour in the task in study 4 in which progress was made without effort.* ***a****. Mean (across subjects and blocks) percentage of high progress choices across trials.* ***b****. Mean (across subjects and blocks) percentage of high progress choices across pressure values. Pressure values where binned in 10 bins.* ***c****. A logistic regression predicting the likelihood of a High Progress Low Reward during Study 4.* ***d****. Mean percentage of goal reached across all blocks in Study 4 (top left) and Study 3 (top right). Mean number of trials before deadline in which the goal was reached across blocks in Study 4 (bottom left) and Study 3 (bottom right).* ***e****. Mean percentage of high progress choices across all blocks, trials and subjects in Study 4 (left) and Study 3 (right). Progress is chosen as or more often when associated to effort than when not (66.45 ± 0.56 % vs 59.94% ± 0.58 %, t(58)= 8.07, p < 0.001).*

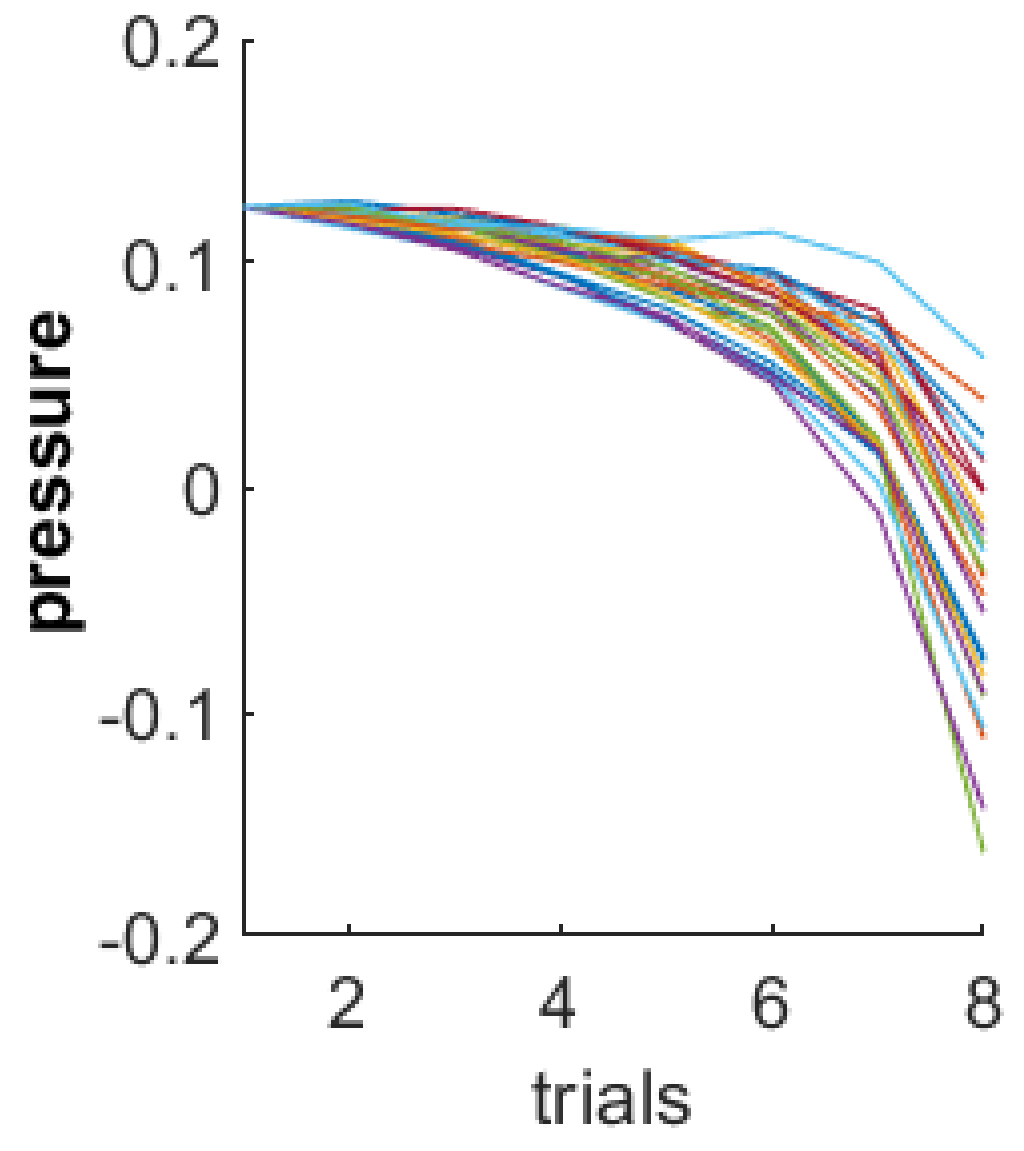

Supplementary Figure 10. *Behaviour in the task in study 1. Average pressure (across successful blocks) over the course of the 8 trials. Each curve represents a participant. Pressure decreases steadily throughout the block when the goal is reached.*

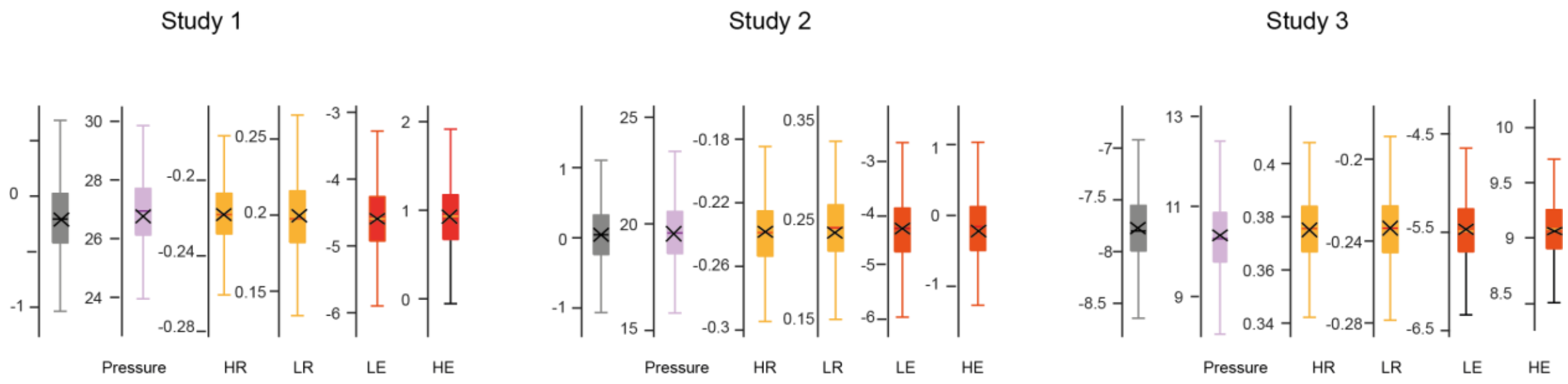

Supplementary Figure 11. *Sensitivity analysis on the effect sizes of the regression to predict choices supporting the statistical power of our main analysis. Boxplot shows the median, lower and upper quartiles, and 5% and 95% percentiles of the simulated distributions. The X shows the effect size from the main dataset (as in Suppl. Figure 3). The medians of the simulations closely capture the effect sizes in all cases. The 95% percentile does not cross 0 for the interventions that have a significant effect on choices. The upper and lower quartiles for all interventions, both significant and non-significant, could be useful for future research to provide the confidence around an expected effect size.*

Supplementary methods

In study 1 prior to the fMRI analysis, we computed correlations between pressure and the other relevant predictors. Pressure was not correlated to effort and reward on offer ($r = -0.01$, $p = 0.19$, for reward, $r = 0.00$, $p = 0.80$, for effort) and mildly correlated with the effort and reward chosen (r = for reward; $r = 0.07$, $p < 0.05$ for effort; $r = -0.25$, $p < 0.001$ for reward) on a trial by trial basis. It was highly correlated with choices (Fig.2b-d; $b = 26.9 \pm 1.2$, $t = 23.031$; $p<0.001$) and mildly correlated with the difference in value between the options ($r = 0.06$, $p <0.001$).

To assess the sensitivity to the predictors of the effect size reported on the regression on choices we simulated 1,000 choice datasets with the same structure and size as the three studies. We did this by sampling participant numbers with replacement the same number of times as the total number of choices, maintaining fixed the reward and effort level for each choice. On each simulated dataset, we then calculated the effect size of the predictors of choices – corresponding to the analysis plotted in Suppl. Figure 3. We then plotted the distribution of effect sizes for each predictors alongside the original effect size (Suppl. Fig.11).

Supplementary Table 1: GLM 1 significant clusters

		Peak MNI coordinates (mm)			
**Region**	**Hemisphere**	***X***	***Y***	***Z***	**Z Value (Peak)**
Pressure					
Supramarginal Gyrus	R	56	-26	46	-7.45
Cingulate Gyrus (RCZp)	R	2	2	36	-5.78
Putamen	R	30	3	-4	-5.56
Inferior Frontal Gyrus	R	54	16	0	-5.10
Superior Frontal Gyrus	L	-30	-2	68	-5.10
Lingual Gyrus	L	24	-52	2	-4.33
Lateral Occipital Cortex	R	46	-62	26	-4.89
Inferior Frontal Gyrus	R	58	14	36	-4.89
Reward					
Occipital Pole	R	18	-96	6	5.89
Lingual Gyrus	L	-28	-60	-4	5.30
Effort (under pressure)					
Paracingulate Gyrus	L	-14	50	18	-4.60
Insula	L	-30	20	-4	-4.41
Middle Frontal Gyrus	L	-44	26	32	-4.19
Effort (without pressure)					
Middle Temporal Gyrus	L	-64	-46	-10	4.81
Middle Frontal Gyrus	R	36	34	36	-6.95
Insula	L/R	±30	20	-6	-5.77
Superior Parietal Lobule	L	-32	-58	52	-4.77
Supramarginal Gyrus	L/R	42	-38	44	-5.34
Caudate	R	10	12	8	-4.89
Frontal Pole	L	-30	46	0	-4.15

Supplementary Table 2: GLM 2 significant clusters

		Peak MNI coordinates (mm)			
**Region**	**Hemisphere**	***X***	***Y***	***Z***	**Z Value (Peak)**
Pressure (offer time)					
Subcallosal Cortex	L	-1	19	-5	-4.15
Lingual Gyrus	R	-8	-81	-6	-4.25
Occipital Pole	L	-14	-98	14	4.93
Middle Temporal Gyrus	R	66	-48	-10	4.95
Pressure (feedback time)					
Fusiform Cortex	R/L	±28	52	-16	-4.97
Lingual Gyrus	L	-10	-64	-6	-4.30
Occipital Cortex	R	26	-64	46	-4.35
Inferior Temporal Gyrus	R	46	-52	-14	-3.99
Advancement (offer time)					
Insula	R/L	±32	26	-4	5.93
Superior Frontal Gyrus	R	4	26	48	5.31
Angular Gyrus	R	52	-48	42	4.92
Middle Frontal Gyrus	R	40	28	42	4.54
Supramarginal Gyrus	L	-42	-46	48	4.37
Frontal Pole	R	30	58	0	4.47
Cingulate Gyrus	R	8	-38	40	4.39
Occipital Pole	L	-36	-90	10	4.18
Occipital Pole	L	-10	-94	8	-4.31
Brain Stem		0	-38	-30	4.78
Middle Temporal Gyrus	R/L	±56	-54	12	-6.69
Lobule Cortex	RL	±4	-4	62	-5.38
Precuneous Cortex	L	-6	-50	54	-4.78
Lateral Occipital Cortex	R	26	-70	54	-4.70
Superior Parietal Lobule	L	-32	-52	52	-4.42
Frontal Pole	R	24	58	24	-5.21

Supplementary Table 3: continued GLM2

Time Left (offer time)					
Fusiform Gyrus	L	-18	-78	-8	5.25
Lateral Occipital Cortex	R	48	-74	30	-5.19
Middle Temporal Gyrus	R	66	-48	0	-4.57
Time Left (feedback time)					
Paracingulate Gyrus	R/L	±8	14	44	-6.12
Postcentral Gyrus	R/L	±42	-20	42	-6.15
Superior Temporal Gyrus	R/L	±58	-28	0	-5.15
Occipital Pole	R/L	±26	-90	-6	-5.83
Brain Stem	R/L	±4	-22	-36	-5.36
Superior Parietal Lobule	L	-40	-40	52	-4.95
Frontal Pole	L	-30	42	32	-4.38
Middle Frontal Gyrus	R	32	6	62	-4.51
Caudate	R	14	14	12	-4.35
Putamen	R	29	-6	-2	-4.28
Inferior Frontal Gyrus	L	-44	24	20	-4.05

Supplementary Table 4: GLM3 significant clusters

Region	Hemisphere	Peak MNI coordinates (mm)			Z Value (Peak)
		X	Y	Z	
Pressure					
Brain Stem	R/L	0	-38	-26	5.48
Subcallosal Cortex	L	-2	0	-12	4.45
Thalamus	R	14	-22	16	-5.95
Paracingulate Gyrus	R/L	±3	11	48	-6.31
Insula	R/L	±36	14	-6.2	-6.31
Postcentral Gyrus	R	52	-22	48	-7.38
Pressure Prediction Error					
Fusiform Gyrus	R/L	±32	-84	-14	5.44
Lobule Cortex - RCZa	L	-2	2	60	5.17
Middle Frontal Gyrus	L	-40	0	54	5.09
Inferior Frontal Gyrus	R/L	±54	14	30	4.83
Postcentral Gyrus	R	36	-34	54	4.59
Fusiform Gyrus	R	22	-84	6	5.39
Posterior Cingulate Gyrus	R/L	±2	-40	18	4.67
Postcentral Gyrus	L	-58	-22	22	4.59
Angular Gyrus	R	48	-50	12	4.50
Middle Temporal Gyrus	R/L	±48	-34	-2	4.37
Lateral Occipital Cortex	R/L	±46	-66	6	4.61
Brain Stem	R/L	0	-24	-28	4.16
Thalamus	L	-8	-16	12	4.30
Mid Cingulate Gyrus	R/L	±4	-22	36	4.16
Precentral Gyrus	R	28	-10	68	4.35
Putamen	L	-20	6	2	4.24

Supplementary Table 5: GLM3 continued

Time Left					
Fusiform Gyrus	L	-28	-80	-6	4.80
Angular Gyrus	R	46	-52	38	-5.54
Insula	R	31	19	-9	-5.59
Reward					
Frontal Pole	R	44	46	24	4.78
Posterior Cingulate Gyrus	R	10	-40	32	4.43
Precentral Gyrus	R/L	±30	-8	50	4.29
Fusiform Gyrus	L	-20	-74	-6	4.73
Lateral Occipital Cortex	L	-26	-82	20	4.83
Superior Frontal Gyrus	R/L	±22	-2	50	4.71
Occipital Pole	R	16	-94	16	5.56
Postcentral Gyrus	L	-42	-24	54	4.41
Superior Parietal Lobule	L	36	-42	42	4.31
Precentral Gyrus	L	-44	0	38	4.26
Effort (under pressure)					
Lingual Gyrus	R	12	-68	-10	4.95
Putamen	R	28	-2	-4	4.72
Paracingulate Gyrus – p32	R	10	50	14	-4.27
Paracingulate Gyrus	R	10	36	26	-4.19
Effort (without pressure)					
Posterior Cingulate Gyrus		0	-46	20	5.55
Middle Temporal Gyrus	R/L	±64	-28	-16	5.13
Frontal Pole	L	-10	66	22	4.69
Frontal Pole	L	-40	38	-8	4.49
Postcentral Gyrus	L	-8	-38	58	4.75
Precentral Gyrus	L	-40	-16	40	4.45
Putamen	R/L	±6	-10	-2	4.44
Lingual Gyrus	R	10	-72	-6	4.76
Opercular Cortex	L	-38	-20	20	4.75
Anterior Cingulate Gyrus – d32	R	6	35	22	-4.10
Paracingulate Gyrus	R	10	30	40	-4.69
Insula	R/L	±32	20	-6	-4.2
Middle Frontal Gyrus	R	34	34	28	-4.34

Supplementary Table 6: GLM4-7 significant clusters

Region	Hemisphere	*X*	*Y*	Z	Z Value (Peak)
Value Difference (signed)					
Lobule Cortex	R	-4	-10	54	4.19
Lingual Gyrus	R/L	0	-66	0	4.76
Anterior Cingulate Gyrus	R/L	±4	36	16	-4.11
Paracingulate Gyrus	L	-10	52	20	-4.13
Value Difference (R-L)					
Precentral Gyrus	L	-32	-26	70	4.46
Paracingulate Gyrus	R/L	±4	36	26	-5.31
Inferior Frontal Gyrus	R	48	24	10	-5.89
Value Difference (signed) - effort discounting model					
Occipital Pole	R/L	±12	-90	-8	6.08
Postcentral Gyrus	L	-40	-24	54	4.36
Supramarginal Gyrus	L	-48	-42	52	4.52
Superior Frontal Gyrus	L	-24	-6	52	4.29
Value Difference (R-L) - effort discounting model					
Anterior Cingulate Gyrus	L	0	32	-2	4.2
Brain Stem	R/L	±2	-38	-26	4.13
Lingual Gyrus	L	-18	-74	-6	3.98
Occipital Pole	L	-10	-98	10	-4.49

Supplementary Table 1-6. BOLD activations in the parametric regressors of our GLMs. Complete list of activations correlating negatively or positively with the single-trial variability. (GLM; mixed effects, $|Z| > 3.8$, corrected). MNI, Montreal Neurological Institute; L, left hemisphere; R, right hemisphere. Coordinates at peak, for bilateral clusters, coordinates of the highest peak.

Supplementary Table 7: Generalised linear regression predicting choices of high effort in study 1

Study 1	Estimate	SE	tStat	pValue
(Intercept)	-0.390	1.56	-0.250	0.802
Pressure	15.2	9.02	1.69	0.0909
$Reward_{Left}$	0.133	0.128	1.04	0.299
$Reward_{Right}$	0.408	0.209	1.95	0.051
$Effort_{Left}$	-2.93	3.85	-0.761	0.447
$Effort_{Right}$	-2.02	2.28	-0.889	0.374
Pressure: $Reward_{Left}$	-0.546	0.478	-1.14	0.25
**Pressure: $Reward_{Right}$**	**1.87**	**0.791**	**2.36**	**0.0183**
**Pressure: $Effort_{Left}$**	**-50.6**	**15.8**	**-3.21**	**<0.001**
**Pressure: $Effort_{Right}$**	**41.6**	**11.8**	**3.52**	**<0.001**
**$Reward_{Left}$:$Reward_{Right}$**	**-0.0359**	**0.011**	**-3.20**	**0.00137**
$Reward_{Left}$: $Effort_{Left}$	-0.164	0.22	-0.738	0.460
$Reward_{Left}$: $Effort_{Right}$	-0.286	0.166	-1.73	0.0842
$Reward_{Right}$: $Effort_{Left}$	0.404	0.352	1.15	0.252
$Reward_{Right}$: $Effort_{Right}$	-0.401	0.26	-1.52	0.128
$Effort_{Left}$: $Effort_{Right}$	6.20	5.23	1.19	0.236

Generalised linear regression predicting choice of the high effort option for Study 1 (fMRI). 4083 observations, 4067 error degrees of freedom. Dispersion: 1 Chi^2-statistic vs. constant model: 1.04e+03, p-value = 5.22e-212.

Supplementary Table 8: Generalised linear regression predicting choices of high effort in study 2

Study 2	Estimate	SE	tStat	pValue
**(Intercept)**	**4.42**	**1.98**	**2.23**	**0.0256**
Pressure	8.95	12.2	0.734	0.463
**$Reward_{Left}$**	**-0.481**	**0.163**	**-2.95**	**0.00322**
$Reward_{Right}$	0.378	0.260	1.45	0.146
**$Effort_{Left}$**	**-10.6**	**4.82**	**-2.198**	**0.0279**
**$Effort_{Right}$**	**-7.70**	**2.91**	**-2.65**	**0.00812**
Pressure: $Reward_{Left}$	-0.28	0.67169	-0.414	0.679
Pressure: $Reward_{Right}$	0.637	1.05	0.609	0.54256
Pressure: $Effort_{Left}$	16.6	21.1	0.784	0.433
Pressure: $Effort_{Right}$	10.7	15.7	0.681	0.496
$Reward_{Left}$:$Reward_{Right}$	-0.0219	0.0136	-1.61	0.107
$Reward_{Left}$: $Effort_{Left}$	-0.112	0.273	-0.409	0.683
**$Reward_{Left}$: $Effort_{Right}$**	**0.595**	**0.203**	**2.94**	**0.00328**
$Reward_{Right}$: $Effort_{Left}$	-0.0236	0.427	-0.0554	0.956
$Reward_{Right}$: $Effort_{Right}$	-0.076	0.316	-0.239	0.811
$Effort_{Left}$: $Effort_{Right}$	8.88	6.40	1.39	0.165

Generalised linear regression predicting choice of the high effort option for Study 2 (behavioural offline). 2238 observations, 2222 error degrees of freedom. Dispersion: 1 Chi^2-statistic vs. constant model: 437, p-value = 1.26e-83

Supplementary Table 9: Generalised linear regression predicting choices of high effort in study 3

Study 3	Estimate	SE	tStat	pValue
(Intercept)	0.404	0.859	0.470	0.638
Pressure	2.46	3.3427	0.7359	0.462
$Reward_{Left}$	-0.084	-0.074	-1.14	0.254
**$Reward_{Right}$**	**0.832**	**0.123**	**6.78**	**<0.001**
**$Effort_{Left}$**	**-5.21**	**2.17**	**-2.40**	**0.016**
**$Effort_{Right}$**	**3.02**	**1.27**	**2.37**	**0.018**
**Pressure: $Reward_{Left}$**	**-0.428**	**0.166**	**-2.57**	**0.010**
**Pressure: $Reward_{Right}$**	**1.26**	**0.27**	**4.59**	**<0.001**
Pressure: $Effort_{Left}$	0.779	5.33	0.146	0.884
Pressure: $Effort_{Right}$	7.14	4.10	1.74	0.082
$Reward_{Left}$:$Reward_{Right}$	-0.055	0.0065	-8.45	**<0.001**
$Reward_{Left}$: $Effort_{Left}$	-0.084	0.127	-0.663	0.508
$Reward_{Left}$: $Effort_{Right}$	0.156	0.0975	1.60	0.110
$Reward_{Right}$: $Effort_{Left}$	-0.11	0.205	-0.555	0.579
**$Reward_{Right}$: $Effort_{Right}$**	**-0.558**	**0.157**	**-3.56**	**<0.001**
$Effort_{Left}$: $Effort_{Right}$	9.93	3.07	3.24	**0.00122**

Generalised linear regression predicting choice of the high effort option for Study 3. 8592 observations, 8576 error degrees of freedom. Dispersion: 1. Chi^2-statistic vs. constant model: 1.12e+03, p-value = 1.56e-228
