# Supplementary material for: Neural and computational mechanisms of effort under the pressure of a deadline
Source: Nat Commun. 2026 Jul 28;17:9169. doi: 10.1038/s41467-026-75987-6 (PMC13522516; doi:10.1038/s41467-026-75987-6)
Supplement: Supplementary file 2 [file 41467_2026_75987_MOESM2_ESM.pdf]

nature portfolio

Corresponding author(s): Michele Andrea Pisauro

Last updated by author(s): Apr 6, 2026

Reporting Summary

Nature Portfolio wishes to improve the reproducibility of the work that we publish. This form provides structure for consistency and transparency in reporting. For further information on Nature Portfolio policies, see our Editorial Policies and the Editorial Policy Checklist.

Statistics

For all statistical analyses, confirm that the following items are present in the figure legend, table legend, main text, or Methods section.

n/a	Confirmed	
☐	☒	The exact sample size (*n*) for each experimental group/condition, given as a discrete number and unit of measurement
☒	☐	A statement on whether measurements were taken from distinct samples or whether the same sample was measured repeatedly
☐	☒	The statistical test(s) used AND whether they are one- or two-sided<br>*Only common tests should be described solely by name; describe more complex techniques in the Methods section.*
☐	☒	A description of all covariates tested
☐	☒	A description of any assumptions or corrections, such as tests of normality and adjustment for multiple comparisons
☐	☒	A full description of the statistical parameters including central tendency (e.g. means) or other basic estimates (e.g. regression coefficient) AND variation (e.g. standard deviation) or associated estimates of uncertainty (e.g. confidence intervals)
☐	☒	For null hypothesis testing, the test statistic (e.g. F, t, r) with confidence intervals, effect sizes, degrees of freedom and P value noted<br>*Give P values as exact values whenever suitable.*
☒	☐	For Bayesian analysis, information on the choice of priors and Markov chain Monte Carlo settings
☒	☐	For hierarchical and complex designs, identification of the appropriate level for tests and full reporting of outcomes
☒	☐	Estimates of effect sizes (e.g. Cohen's d, Pearson's r), indicating how they were calculated

Our web collection on statistics for biologists contains articles on many of the points above.

Software and code

Policy information about availability of computer code

Data collection	We acquired the fMRI data using a 7T Siemens Magnetom MRI scanner. Visual stimuli were generated using MATLAB 2012. Choice data were acquired through MATLAB whereas force data in the effort experiment were acquired through a hand-held dynamometer (SS25LA, BIOPAC Systems, USA) and its associated software (Acqknowledge v.5)
Data analysis	Pre-processing and analysis of the fMRI data was performed using the FMRIB's Software Library (Functional MRI of the Brain, Oxford, UK). Analysis and modelling of behavioural data was performed through MATLAB code developed in the lab deposited in the Open Science Framework project https://osf.io/5kjgb/

For manuscripts utilizing custom algorithms or software that are central to the research but not yet described in published literature, software must be made available to editors and reviewers. We strongly encourage code deposition in a community repository (e.g. GitHub). See the Nature Portfolio guidelines for submitting code & software for further information.

Data

Policy information about availability of data

All manuscripts must include a data availability statement. This statement should provide the following information, where applicable:

- Accession codes, unique identifiers, or web links for publicly available datasets
- A description of any restrictions on data availability
- For clinical datasets or third party data, please ensure that the statement adheres to our policy

The fMRI statistical maps from analyses and behavioural data generated in this study have been deposited in the Open Science Framework project https://osf.io/5kjgb/. The raw fMRI data are protected and are not available due to data privacy laws. All data and code will be made available at the time of publication.

Research involving human participants, their data, or biological material

Policy information about studies with human participants or human data. See also policy information about sex, gender (identity/presentation), and sexual orientation and race, ethnicity and racism.

Reporting on sex and gender	We recruited 125 healthy young participants across three studies. In study 1 (N = 30, 16 female, 14 male, age range 18-40). In study 2, (N = 15 participants, 8 female, 7 male, age range 18-40). In study 3, (N = 40, 11 female 29 male, age range 20-45). In study 4, (N= 30, 12 female 17 male, 1 prefer not to say, age range 18-45). This information refer to their self-declared gender.
Reporting on race, ethnicity, or other socially relevant groupings	We did not recruit participants according to their race or ethnicity nor we recorded this information or controlled for it in our analysis.
Population characteristics	All subjects had normal or corrected-to-normal vision and reported no history of psychiatric, neurological or major medical problems, and were free of psychoactive medications at the time of the study.
Recruitment	All participant for study 1 and 2 were recruited from the participants' database of the department of Experimental Psychology at the University of Oxford. For study 3 and 4 participants were recruited from online platform Prolific.com and performed a version of the task online on Gorilla.sc, an online experiment builder. This ensured a wider sample of UK participants
Ethics oversight	All studies were approved by The University of Oxford Central Research Committee. Informed consent was obtained from all participants.

Note that full information on the approval of the study protocol must also be provided in the manuscript.

Field-specific reporting

Please select the one below that is the best fit for your research. If you are not sure, read the appropriate sections before making your selection.

☐ Life sciences ☒ Behavioural & social sciences ☐ Ecological, evolutionary & environmental sciences

For a reference copy of the document with all sections, see nature.com/documents/nr-reporting-summary-flat.pdf

Behavioural & social sciences study design

All studies must disclose on these points even when the disclosure is negative.

Study description	Participants took part in an effort-based task while quantitative behavioural and fMRI data were collected.
Research sample	We recruited 125 healthy young participants across three studies. In study 1 (N = 30, 16 female, 14 male, age range 18-40). In study 2, (N = 15 participants, 8 female, 7 male, age range 18-40). In study 3, (N = 40, 11 female 29 male, age range 20-45). In study 4, (N= 30, 12 female 17 male, 1 prefer not to say, age range 18-45). For the first two studies the recruitment was from the student population of the University of Oxford, and the wider community of the city of Oxford. For study 3 and 4 participants were recruited from online platform Prolific.com and performed a version of the task online on Gorilla.sc, an online experiment builder. This ensured a wider sample of UK participants.
Sampling strategy	The sample size for study 1 (N= 30) was chosen based on previous effort-based studies reporting strong effect sizes obtained with 3T fMRI scanners at N = 35 (ref 10,27) and considering the up two times higher signal to noise of 7T scanners (ref82). For study 2 sample size was based on a power analysis of the effect size of pressure that a smaller sample was sufficient to replicate. The sample size for study 3 was chosen based on related previous studies assessing the relationship between fatigue scores with effort-based choices (ref 47) for N = 40. For study 4, where there was no effort nor fatigue, we matched the sample size of study 1.
Data collection	Across all studies, the same basic design was used to examine how the willingness to exert effort to obtain rewards changes over trials under the pressure to reach a goal by a deadline. All studies comprised an initial calibration phase to establish their maximum voluntary contraction (MVC – study 1 and 2) on a handheld dynamometer, or maximum number of mouse button clicks (MVNC -

	study 3) followed by a training phase, in which participants were familiarised with each level of effort that they would be required to perform during the rest of the experiment. This was followed by an effort-discounting task to assess their effort sensitivity in a more standard paradigm where there was no goal, and the deadline task, where they had to complete goals. In study 1, the deadline task was performed within the fMRI scanner.
Timing	05/2019- 03/2020 and 03/2021 - 03/2022
Data exclusions	Four participants (2 in study 2, 2 in study 3) were excluded due to a failure to follow task instructions or for low performance in the task (the exclusion criterion was met if the participant didn't succeed in at least 10 blocks out of 30).
Non-participation	No participants declined participation.
Randomization	There were no different experimental groups.

Reporting for specific materials, systems and methods

We require information from authors about some types of materials, experimental systems and methods used in many studies. Here, indicate whether each material, system or method listed is relevant to your study. If you are not sure if a list item applies to your research, read the appropriate section before selecting a response.

Materials & experimental systems

n/a	Involved in the study
☒	☐ Antibodies
☒	☐ Eukaryotic cell lines
☒	☐ Palaeontology and archaeology
☒	☐ Animals and other organisms
☒	☐ Clinical data
☒	☐ Dual use research of concern
☒	☐ Plants

Methods

n/a	Involved in the study
☒	☐ ChIP-seq
☒	☐ Flow cytometry
☐	☒ MRI-based neuroimaging

Plants

Seed stocks	*Report on the source of all seed stocks or other plant material used. If applicable, state the seed stock centre and catalogue number. If plant specimens were collected from the field, describe the collection location, date and sampling procedures.*
Novel plant genotypes	*Describe the methods by which all novel plant genotypes were produced. This includes those generated by transgenic approaches, gene editing, chemical/radiation-based mutagenesis and hybridization. For transgenic lines, describe the transformation method, the number of independent lines analyzed and the generation upon which experiments were performed. For gene-edited lines, describe the editor used, the endogenous sequence targeted for editing, the targeting guide RNA sequence (if applicable) and how the editor was applied.*
Authentication	*Describe any authentication procedures for each seed stock used or novel genotype generated. Describe any experiments used to assess the effect of a mutation and, where applicable, how potential secondary effects (e.g. second site T-DNA insertions, mosiacism, off-target gene editing) were examined.*

Magnetic resonance imaging

Experimental design

Design type	Task and event related.
Design specifications	Participants performed 30 blocks of 8 trials each.
Behavioral performance measures	We recorded button presses indicating choices and force data for exertion of physical effort. Participants responded in a large majority of trials (response rate >95%)

Acquisition

Imaging type(s)

functional MRI

Field strength

7T

Sequence & imaging parameters

We collected functional Echo-Planar Imaging (EPI) data using a 32-channel head coil (repetition time: 1.5 s; echo time: 0.73 ms; number of slices: 93; number of voxels: XX × YY; in-plane resolution: 1.5 × 1.5 mm; slice thickness: 1.5 mm; flip angle: 66°). Altogether, we collected 1840 volumes each, corresponding to thirty blocks of 8 trials each for a total of 240 trials in the main experimental task.

Area of acquisition

whole brain

Diffusion MRI ☐ Used ☒ Not used

Preprocessing

Preprocessing software

Pre-processing of our data was performed using the FMRIB's Software Library (Functional MRI of the Brain, Oxford, UK) and included: head-related motion correction, slice-timing correction, high-pass filtering (>100s), and spatial smoothing (with a Gaussian kernel of 8mm full-width at half maximum).

Normalization

To register our EPI image to standard space, we first transformed the EPI images into each individual's high-resolution space with a linear six-parameter rigid body transformation. We then registered the image to standard space (Montreal Neurological Institute, MNI) using FMRIB's Non-linear Image Registration Tool with a resolution warp of 10mm.

Normalization template

MNI152_T1_2mm_brain

Noise and artifact removal

The preprocessing included head-related motion correction, slice-timing correction, high-pass filtering (>100s), and spatial smoothing (with a Gaussian kernel of 8mm full-width at half maximum). We also acquired a B0 map using a multi-shot gradient echo sequence which was subsequently used to correct for distortions in the EPI data due to B0 inhomogeneities (echo time: 2.3ms; delta echo time: 5ms; isotropic resolution: 3mm; matrix: 68 × 68 × 32; repetition time: 383ms; flip angle: 90°).

Volume censoring

we didn't censor volumes

Statistical modeling & inference

Model type and settings

We performed whole-brain statistical analyses of functional data using a multilevel approach within the generalized linear model (GLM) framework, as implemented in FSL through the FEAT module: Y = Xβ + ε = β1X1 + β2X2 + ... + βNXN + ε where Y is a T ×1(T time samples) column vector containing the times series data for a given voxel, and X is a T × N (N regressors) design matrix with columns representing each of the psychological regressors convolved with a hemodynamic response function specific for human brains. β is a N × 1 column vector of regression coefficients and ε a T × 1 column vector of residual error terms. Using this framework we initially performed a first-level fixed effects analysis to process each individual experimental run which were then combined in a second level mixed-effects analysis (FLAME 1 + 2) to combine data across subjects, treating participants as a random effect. For all analysis, we performed a cluster inference using a cluster-defining threshold of |Z| > 3.8 with a FWE-corrected threshold of P = 0.001.

Effect(s) tested

We performed the GLMs highlighted below.
All GLMs included three unmodulated stick function regressors: (1) at the onset of the stimuli, (2) at the time of the choice (3) at the time the feedback appeared on the screen. Trials without pressure are defined as trials where pressure is negative according to equation 1, that is, when the goal had been already reached.
GLM 1: Included a parametric regressor modulated by the trial-by-trial pressure experienced by participants at the time of the offer one modulated by the reward and effort (separately for trials with or without pressure) chosen at time of choice.
GLM 2: Included parametric regressors for pressure, advancement and time left at time of choice and of feedback.
GLM 3: Included parametric regressors for time left at time of choice, for reward and effort (divided by trials with or without pressure) at time of choice, and pressure and pressure prediction error at time of feedback
GLM 4: Included parametric regressors for pressure at time of offer, signed difference in value (as computed by the model) between the higher and lower effort option at time of choice, and advancement and time left at time of feedback.
GLM 5: this GLM was identical to GLM 4 substituting the signed difference in value with an unsigned absolute difference in value between the two options.
GLM 6 and 7: identical to GLM 4 and 5 but with the value computed by a standard effort discounting model (where effort is a cost).

Specify type of analysis: ☐ Whole brain ☐ ROI-based ☒ Both

Anatomical location(s)

We drew masks of the cingulate regions based on anatomical maps (ref 36) of the cingulate cortex (with a threshold of 12) and of putamen following (ref 85).

Statistic type for inference

(See Eklund et al. 2016)

For all analysis, we performed a cluster inference using a cluster-defining threshold of |Z| > 3.8 with a FWE-corrected threshold of P = 0.05.

Correction

FWE-corrected threshold of P = 0.05

Models & analysis

n/a	Involved in the study
☐	☒ Functional and/or effective connectivity
☒	☐ Graph analysis
☒	☐ Multivariate modeling or predictive analysis

Functional and/or effective connectivity

we extracted time-series data from individual clusters in 5 cingulate regions and two subdivisions of Putamen, which served as a seed region (that is, the physiological regressor—PHY) for a PPI analysis. This analysis was primarily designed to investigate the potential interaction of the area encoding pressure and pressure prediction error. We constructed our psychological (PSY) task regressor as a parametric regressor boxcar regressor with a step function in the interval between stimulus onset and response time or for 1,5 second following the presentation of the feedback.
