# Supplementary material for: Neural and computational mechanisms of effort under the pressure of a deadline
Source: Nat Commun. 2026 Jul 28;17:9169. doi: 10.1038/s41467-026-75987-6 (PMC13522516; doi:10.1038/s41467-026-75987-6)
Supplement: Supplementary file 3 [file 41467_2026_75987_MOESM3_ESM.pdf]

nature portfolio

Peer Review File

Neural and computational mechanisms of effort under the pressure of a deadline

Corresponding Author: Dr M Andrea Pisauro

This manuscript has been previously reviewed at another journal. This document only contains information relating to versions considered at Nature Communications.

This file contains all reviewer reports in order by version, followed by all author rebuttals in order by version.

Version 0:

Reviewer comments:

Reviewer #4

(Remarks to the Author)
Pisauro and colleagues present a study (across 4 experiments) examining the neural and computational mechanisms of effort under the pressure of a deadline. They hypothesize that while effort costs are typically aversive, that the pressure of a deadline determines whether physical effort is perceived as an avoided cost verses something to be sought. They find that participants choose higher effort options (relative to high reward) as a function of the pressure being caused by the deadline and the ease of progressing in the task to complete the goal. They develop a computational model that parametrizes pressure sensitivity and find that it is a predictor of higher effort options being sought and goal completion more generally. They also observe a region of rostral mid cingulate cortex and putamen associated with pressure associated with reaching a goal and observe distinct portions of ACC signaling effort with and without pressure (rostral ACC vs. dorsal ACC).

This is an innovative study with an interesting paradigm, computational modeling, and neuroimaging that probes the effects of pressure, reward, and effort in a decision-making paradigm. The results are interesting and of relevant to the field, however I have several comments that moderate my enthusiasm for the manuscript in its current form. My comments are detailed below, and my intention is providing suggestions that I think will enhance the overall impact of the paper.
Major comments
- I am overall concerned about the generalizability of the effects when reported by individual study, as the sample sizes across all 4 studies are relatively small. (Study 1: N=30, Study 2: N=15, Study 3: N=40, Study 4: N=40). This is especially concerning as many of the patterns in studies 1 and 3 are not replicated in study 2, and it is not clear whether the lack of effects is due to insufficient sample size. It would be helpful to include a power analysis to show whether the experiments were sufficiently powered (either via sample size or via sufficient trials of each condition) to generate the expected effects. The tiny sample size for the behavioral study (N=15) is especially concerning. It is not clear why some of the analyses are collapsed across studies while others are not.

- It is not clear that the evidence fully supports the author's claim about people prioritizing high effort options (starting at line 188). First, this effect is not reliably produced across all studies (e.g., no effect in study 2). Second, the effect sizes for the choosing the high effort option are relatively small (at least compared to the pressure effect). Finally, given that there is a significant interaction between pressure and choosing the high effort option, this would weaken any interpretation about the high effort option. Instead, it would be conservative and statistically accurate to highlight how pressure was the most significant predictive but other factors were also predictive but not as strong (as stated in Figure 2d). Inclusion of a hierarchical logistic regression analyses would demonstrate this point more clearly. It would also be helpful to include in the logistic regressions the interaction between pressure and these variables (pressure, reward, effort), which would clarify to what extent the choice is driven by the main effect of these variables or instead the interaction between them (which are suggested by Figures 2a + 2b).

- There is not compelling evidence that deadline pressure parameter is linked to reach goals or deadlines in everyday life, as this association was only observed in study 3 with a n=40 and it is not clear that this sample is sufficiently powered. Can this correlation analysis be performed by collapsing all the data across multiple studies? Additionally, it is confusing why that

data is included (or not) in Figures 3f and 3h (there seem to be only 30 reported?). Given concerns about power and that this is the same(?) task across studies, it is not clear why the data were not combined for the model fitting and reported associations between pressure sensitivity (k) and goal completion metrics.

- The dissociation between rostral vs. dorsal ACC for pressure vs. no pressure trials is compelling. I wonder to what extent the "effort no pressure" could be re-considered as a "bias towards pursuing high reward". That is, the contextual difference of having pressure to complete a goal might require critical evaluation of the choices, whereas without the pressure the individual may be deciding which action would maximize reward (consistent with past work in this domain). This does not refute the current finding or interpretation, but I think it could be helpful to contextualize this finding in light of the broader extant literature and should be clarified in the Discussion.

- It is not clear that Choice Value analyses (high effort vs. low effort option) does not actually reflect the subjective evaluation of effort accurately, as effort offer is confounded with reward value (high reward was also yoked with low effort, low reward was always yoked with high effort). Are these analyses controlling for reward amount? Perhaps I am misunderstanding this figure, but it is not clear whether the analyses in Figure 4d reflect differences in reward value or effort value – if effort is higher than would we not anticipate activation to correspond to high effort / low value options? Clarification of these analyses and the corresponding figure is important to substantiate the author's claims about neural activation to choice value. It is not clear that the evidence in the current form how these choices under pressure relate to subjective value of effort-based choice.
Minor comments
- Line 199, this sentence is difficult to parse: "Secondly, as outlined above we showed that people sought the higher effort option more often the more it was higher in effort and also were more likely to choose the lower effort option when this lower effort was closer to the higher level." What do the authors mean by that people choosing the lower option level when lower effort was closer to the higher level? I cannot make sense of this sentence – it would be helpful to clarify the various decision option labels.
- Line 469 – "we replicated in three experiments the finding that people seek higher effort behaviors, that are associated with lower immediate rewards" -- This is not accurate, this should be revised to reflect the finding about pressure and the claims about high effort effects only should be tempered considering the evidence presented in the manuscript.
- There are numerous typos in the Discussion, please check for for grammatical errors.
- The discussion section overall is quite lengthy and longwinded. This could be streamlined to focus on the key points of the paper, as well as contextualize the authors' findings (which are exciting!) and how they update our understanding of these neural and computational mechanisms of effort-based decision making instead of primarily pontificating on the paradoxical nature of effort or other factors beyond the scope of the study (i.e., procrastination). WRT the past theories on ACC/MCC function, rather than listing all the past theories, it may be useful to clarify to what extent the current findings are consistent or are inconsistent with past theory/data, as it is well known that this region is heterogeneous and interpretations idiosyncratic based on the researcher and experiment. Trimming this section would help the reader better understanding the novelty of these findings.
- This paper mentions ultra-high-field MRI as a feature of this study – but did not discuss how this would benefit over regular MRI. Is the main advantage that there is better tSNR in the subcortical regions (e.g., putamen)? Would be helpful to mention how this was an advantage in this study.

In addendum to my review, I have been requested to comment on the response to the author's rebuttal to R2 + R3 critiques.

Overall, I find that the authors are responsive to these reviewers' comments. However, I do have some minor concerns, detailed below.

The authors state "Without a longer-term goal and pressure to reach this by a deadline, people would always choose more reward and less effort", which is may not be entirely accurate as this hinges on the normative assumption that on a given decision the receipt of monetary reward is endogenously more valuable than exerting effort. For instance, this would not be true in the case where people entirely discount the value of money (or whatever reward) or may find intrinsic value in selecting the high-effort option.

The authors make the following claim: "Typically making this progress requires effort, both in our task and in everyday life, so people choose higher effort when under high deadline pressure to make as much progress as possible and thus ensure they reduce the pressure and complete goals in time."

I do have a concern about this claim. This is likely true in the context of this study since the amount of effort required is not disproportionately larger than the amount of expected reward/benefit for exerting that effort. For instance, if an individual is completely incapable of exerting the force required, then they would never make that choice. I think it would help to include some language limiting the scope of when higher effort is selected under deadline pressure, stating the caveat that these effects likely are driven when effort is proportional (or comparable to) expected reward. While investigating the boundary conditions of when effort is no longer worthwhile or beneficial in the progress-towards-goal context is outside of the context of the current study, stating these experimental limitations is important for the reader to understand how generalizable these effects are (or not) to everyday life.

(Remarks on code availability)
I was not able to review the code as the repository was set to private required to request access to review. I did not do this because it would have unblinded my review. It would be helpful to change the settings of the OSF to allow for code review by a general audience, to evaluate the reproducibility of the code generated by this project.

Reviewer #5

(Remarks to the Author)
For context, I have been asked to evaluate whether this revision has addressed the previous reviewers' outstanding concerns.

With regards to the comments to the framing of the results—in particular, the “effort adds value” interpretation—I believe the language used in the revision now appropriately interprets these results as: deadline pressure can induce effort expenditure (even for small reward amounts).

However, I am not convinced that the results of Experiment 4 (where no effort was required to advance goal progress) demonstrates anything meaningful over and above the existing studies. That is, I simply cannot see how the result that subjects are slightly less likely to choose options which confer greater progress (fig 9E) when there is no effort required as support for the idea that effort imbues goal progress with value. First, the key comparison depicted in Fig 9E is made between experiments, rather than conditions within an experiment, in the absence of random assignment (which, in my view, precludes the use of inferential statistics like t-tests across experiments). The magnitude of this difference is small, and I would expect that, across identical replications of this choice task, this overall preference would move slightly around between experiments simply owing to noise. So what conclusion can be drawn on the basis of comparing these two different proportions that were obtained at different times (e.g. study 3 and study 4) with different samples and different designs? According to psychological research methods orthodoxy: very little.

Finally, given that this study specifically examines physical effort I wondered why the authors used such a broad “effort” framing instead of a more specific framing about the type of effort considered here (physical). This framing used throughout the paper struck me as rather underconstrained because it gives the impression that the findings concerning effort perception or fatigue pertain to effort in general—but in reality these findings only speak to physical effort.

(Remarks on code availability)

Version 1:

Reviewer comments:

Reviewer #4

(Remarks to the Author)
The authors did a fantastic job of thoroughly addressing all my comments, which I greatly appreciate (and acknowledge is a challenge with multiple rounds of review). I am happy to recommend that the manuscript for acceptance. Congrats on a comprehensive paper!

P.S. There is a minor typo on page 8 (not sure what supplementary figure X is referring to).

(Remarks on code availability)

Reviewer #5

(Remarks to the Author)
I am by and large satisfied with the authors' revisions made in response to my concerns and I am happy to recommend publication.

(Remarks on code availability)

Open Access This Peer Review File is licensed under a Creative Commons Attribution 4.0 International License, which permits use, sharing, adaptation, distribution and reproduction in any medium or format, as long as you give appropriate credit to the original author(s) and the source, provide a link to the Creative Commons license, and indicate if changes were made.
In cases where reviewers are anonymous, credit should be given to 'Anonymous Referee' and the source.
The images or other third party material in this Peer Review File are included in the article's Creative Commons license, unless indicated otherwise in a credit line to the material. If material is not included in the article's Creative Commons license and your intended use is not permitted by statutory regulation or exceeds the permitted use, you will need to obtain permission directly from the copyright holder.
To view a copy of this license, visit https://creativecommons.org/licenses/by/4.0/

REVIEWER COMMENTS:

Reviewer #4 (Remarks to the Author):

Pisauro and colleagues present a study (across 4 experiments) examining the neural and computational mechanisms of effort under the pressure of a deadline. They hypothesize that while effort costs are typically aversive, that the pressure of a deadline determines whether physical effort is perceived as an avoided cost verses something to be sought. They find that participants choose higher effort options (relative to high reward) as a function of the pressure being caused by the deadline and the ease of progressing in the task to complete the goal. They develop a computational model that parametrizes pressure sensitivity and find that it is a predictor of higher effort options being sought and goal completion more generally. They also observe a region of rostral mid cingulate cortex and putamen associated with pressure associated with reaching a goal and observe distinct portions of ACC signaling effort with and without pressure (rostral ACC vs. dorsal ACC).

This is an innovative study with an interesting paradigm, computational modeling, and neuroimaging that probes the effects of pressure, reward, and effort in a decision-making paradigm. The results are interesting and of relevant to the field, however I have several comments that moderate my enthusiasm for the manuscript in its current form. My comments are detailed below, and my intention is providing suggestions that I think will enhance the overall impact of the paper.

We thank the reviewer for the positive assessment of our work and for the constructive comments. We agree that the reviewers' comments have helped us to improve the overall impact of the work and believe we have now addressed their concerns.

Major comments

- I am overall concerned about the generalizability of the effects when reported by individual study, as the sample sizes across all 4 studies are relatively small. (Study 1: N=30, Study 2: N=15, Study 3: N=40, Study 4: N=40). This is especially concerning as many of the patterns in studies 1 and 3 are not replicated in study 2, and it is not clear whether the lack of effects is due to insufficient sample size. It would be helpful to include a power analysis to show whether the experiments were sufficiently powered (either via sample size or via sufficient trials of each condition) to generate the expected effects. The tiny sample size for the behavioral study (N=15) is especially concerning. It is not clear why some of the analyses are collapsed across studies while others are not.

It is not clear that the evidence fully supports the author's claim about people prioritizing high effort options (starting at line 188). First, this effect is not reliably produced across all studies (e.g., no effect in study 2).

We thank the reviewer for the comment. To address it we have modified text in a number of places, provided a bootstrapping analysis (and additional supplementary figure) and clarified how results are analysed separately for each study.

In regards to the collapsed analyses, we are a little unsure to what the reviewer refers to. None of the main statistical analyses presented in the manuscript were collapsed across studies but were run on the individual studies separately. We have now ensured that all analyses are attributed to the correct study in the text and modified the caption of figure 3 to ensure this is clear.

In regards to the generalizability of the effects, while we appreciate the reviewer's concern, we think it is a strength of the paper that the key results, regarding the effect of deadline pressure, are replicated across studies one to three, with many results also replicated in study 2.

We appreciate the concern that the effect of effort being positively valued is replicated in study 3 but not in study 2. However, there are a number of reasons why our interpretations remain sound, even with that specific effect not being fully replicated across all studies, and also we provide new analyses to demonstrate that the effects in study 1 and 3 are robust.

Firstly, it's important to note that participants do choose the higher effort option more often in study 2 than would be expected if they were effort-discounting - this is shown in the main effect of pressure being highly significant. This indicates that participants are choosing the higher effort option when pressure is higher, when they preferred to avoid it in a task with no goals. This is consistent with a shift in the valuation of effort to being positive - even if it is not as strong evidence as people choosing the higher effort option more often when it was highest in effort.

Secondly, we note that the modelling results - in which pressure increases the value of effort - all replicated including in study 2. These results, in addition to the statistical ones, indicate that pressure is increasing the value of effort and this is driving choices in the task.

Thirdly, we completed a new bootstrapping analysis (this was completed rather than post-hoc power analyses at the request of the editor) to assess the robustness and generalizability of the effect sizes in our regression to outliers and perturbations of our dataset. In order to do so, we simulated 1,000 behavioural datasets with the same structure and number of trials as our real datasets via bootstrapping. We did this by sampling participant numbers with replacement and regressing choices with the same predictors as in fig.2d and suppl.fig.3. We then plotted the distribution of effect sizes for all predictors in the three experiments. As it can be seen in the new supplementary figure 11 the medians of the simulations closely capture the effect sizes in all cases, suggesting that our sample size was in all cases sufficient to capture the likely strength of these effects. This indicates that the results of pressure and effort being chosen more often when higher in effort are not by chance, but are robust effects that generalise based on the sample sizes we have collected.

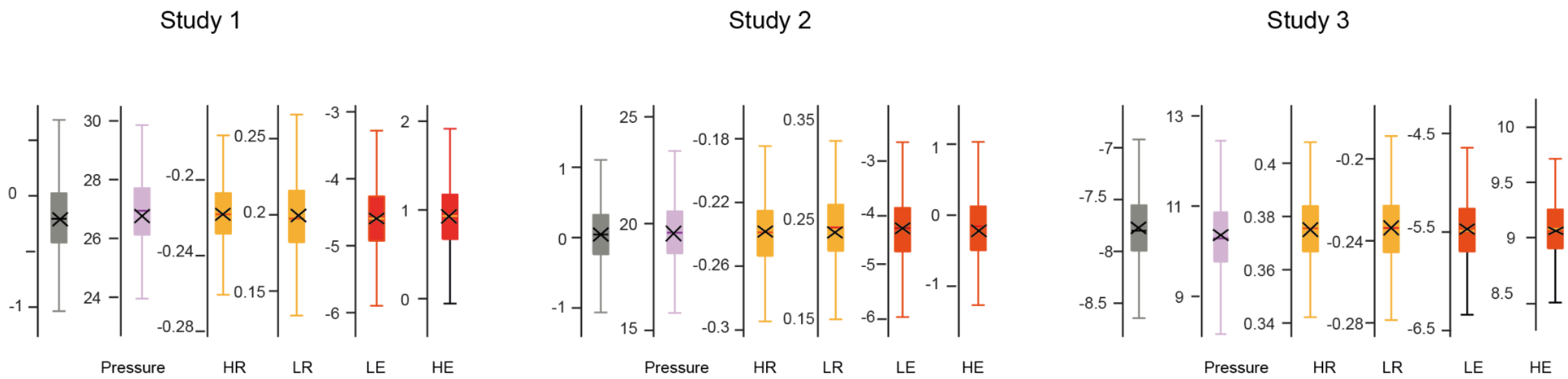

Suppl. Fig.11

- Second, the effect sizes for the choosing the high effort option are relatively small (at least compared to the pressure effect). Finally, given that there is a significant interaction between pressure and choosing the high effort option, this would weaken any interpretation about the high effort option. Instead, it would be conservative and statistically accurate to highlight how pressure was the most significant predictive but other factors were also predictive but not as strong (as stated in Figure 2d). Inclusion of a hierarchical logistic regression analyses would demonstrate this point more clearly. It would also be helpful to include in the logistic regressions the interaction between pressure and these variables (pressure, reward, effort), which would clarify to what extent the choice is driven by the main effect of these variables or instead the interaction between them (which are suggested by Figures 2a + 2b).#

We thank the reviewer for this comment and appreciate their points.

We agree with the reviewer that pressure is the most significant predictor and we have attempted to make this clear in the manuscript in multiple places. However, this is not to say that the effect of the high effort on choice is not important, significant and meaningful. The statistical models we included within the logistic regressions were selected through model comparison. These comparisons included models in which only pressure was included and not the level of effort. The models without the offered effort of each offer were poorer fits to the data, indicating that the offered levels of effort were needed in the models to best explain choice behaviour - this suggests that they do meaningfully contribute to the choices and across two studies. The computational modelling results also demonstrate that effort increased value, also selected through model comparison and these results replicated across all three studies.

In addition, there is some caution to overinterpret the size of the effects relative to each other in too strong a manner due to the nature of the task design. This is due to the fact that the high effort effort level only varied across two levels and was used as a predictor on all trials, regardless of whether the goal was near or had been completed. However, on the final few trials participants are not picking the high effort option and thus this is reducing the effect size of these predictors. (i.e. if a goal has been completed, the effort level of the high effort option will become redundant as people will not pick it, reducing the potential effect size of the variable) In contrast the pressure variable is highly continuous and still is meaningful when a goal is close to being completed (i.e. if a goal has been nearly completed, people will choose the low effort option as pressure approaches zero). This means that while it is clear pressure is the most important factor for driving choices in the study, comparing its

effect size to the other variables in the statistical models should be treated with some caution.

To answer the question of whether pressure drives decision in itself or through the interaction with the effort level, in the model comparison of different regression models, we included both models with just individual predictors and models with their interactions. The regression model we report about in the manuscript had the lowest BIC in this model comparison, suggesting it provides a more parsimonious explanation of the choice data than the model with the full interactions.

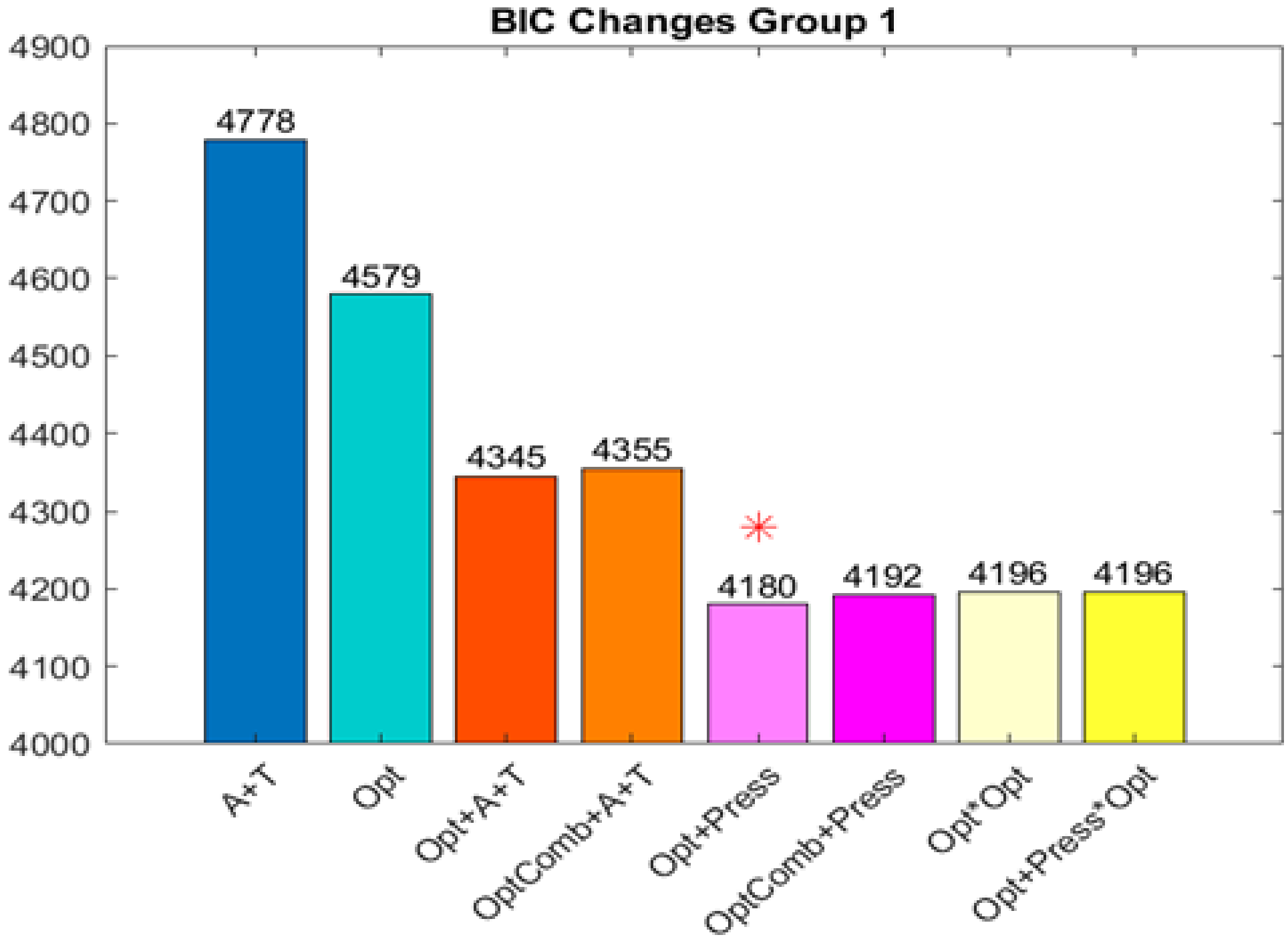

*Model comparison analysis of different regression models. Labels express the variable used as regressors. Opt stands for the options in terms of the two rewards and two effort levels. A stands for advancement towards the goal. T for trial number. Interaction terms are denoted by . Both models including interactions (Opt*Opt and Opt + Press*Opt) have a higher BIC of the model with just the options and pressure, that we report in the manuscript.*

We did not intend this analysis as strong evidence against the presence of an interaction. Indeed, when including the interaction terms in the regression, pressure had significant interactions with the effort levels of both options (as expected increasing the likelihood they were chosen proportionally to both pressure and the effort level) and with the reward of the high effort low reward option:

pressure:reward_left	*b* = -0.54597±0.47784	t= -1.1426	p =0.25321
**pressure:reward_right**	***b* = 1.8652 ±0.79061**	**t= 2.3591**	**p= 0.018319**
**pressure:effort_left**	***b* = -50.556±15.757**	**t=-3.2085**	**p<0.001**
**pressure:effort_right**	***b* = 41.566±11.821**	**t= 3.5163**	**p<0.001**

While we caution against interpreting this strongly, because including the interaction terms did not improve the model fit, our modelling results also suggest that pressure has a significant influence on choices by modulating the values of the two options and this

modulation is driven by the interaction of pressure and effort, on both options, suggesting that effort becomes valuable only under pressure.

Therefore, we agree with the reviewer that these points need to be considered better in the manuscript and we have now changed the wording of several sections of text to make these points clearer in addition to highlighting how our statistical models had been selected.

- There is not compelling evidence that deadline pressure parameter is linked to reach goals or deadlines in everyday life, as this association was only observed in study 3 with a n=40 and it is not clear that this sample is sufficiently powered. Can this correlation analysis be performed by collapsing all the data across multiple studies? Additionally, it is confusing why that data is included (or not) in Figures 3f and 3h (there seem to be only 30 reported?). Given concerns about power and that this is the same(?) task across studies, it is not clear why the data were not combined for the model fitting and reported associations between pressure sensitivity (k) and goal completion metrics.

We thank the reviewer for this comment. We did not collect responses to the FAS questionnaire in study 1 and 2 which were conducted in person, we are therefore not able to perform any collapsed analyses as suggested. We have therefore clearly specified in the methods (line 870) and in the results that everyday fatigue data were only collected in study 3 and added a caveat in the discussion (lines 575-585) clarifying the need for future experiments that now reads:

This variability was captured by the pressure sensitivity parameter within our model, which in turn was associated with daily levels of fatigue ***collected in study 3****. Previous work has shown that higher levels of fatigue are associated with a reduced willingness to exert effort for reward, putatively due to an increased cost of effort.* ***While this analysis was only performed in study 3 and therefore warrants replication, it suggests that feelings of fatigue might reduce people's sensitivity to goals pressure****. Moreover, this suggests that our computational model may be fruitful for understanding variability in motivation, and disorders associated with reduced fatigue.* ***Future work can probe the strength and directionality of the link between fatigue and diminished sensitivity to pressure across different tasks****.*

As requested by the reviewer, we have also run the correlation between the model parameters and the goal completion metrics across studies (new suppl. Figure 5). Additionally we have now clarified in the caption of figure 3 that only data from study 1 are included there.

We reran correlations between the model parameter and different aspects of the task by collapsing the data across all subjects of the three tasks. We found that, consistently with what we observed in each individual studies, and reported in the new suppl.fig.5., that our pressure sensitivity parameter was significantly correlated with the proportions of goals reached (ρ = 0.28; $p<0.05$) and anticorrelated with the average trial at which participants reached their goals (ρ = -0.26; $p<0.05$).

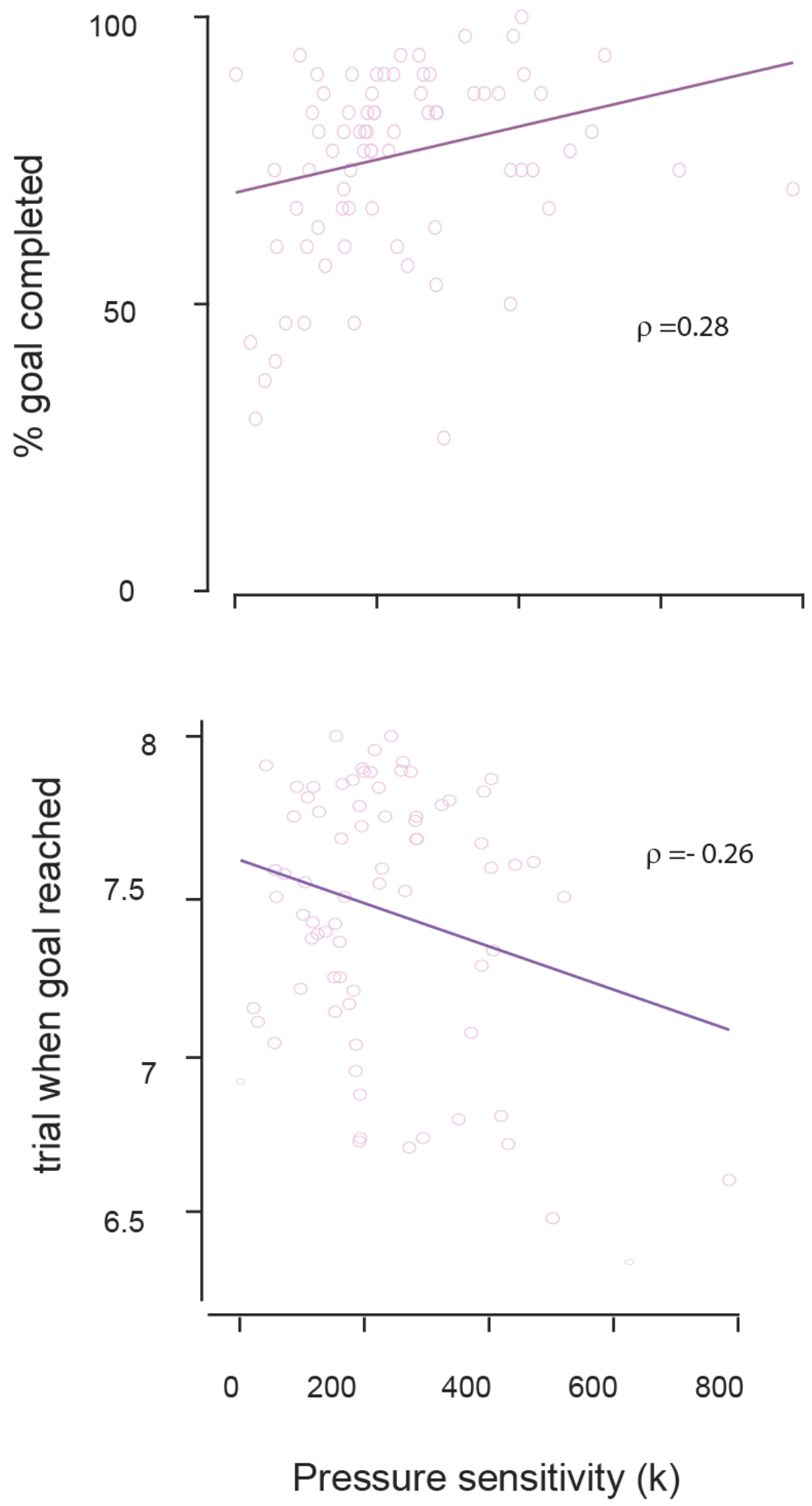

100
50
0
% goal completed
ρ =0.28
8
7.5
7
6.5
trial when goal reached
ρ =- 0.26
0
200
400
600
800
Pressure sensitivity (k)

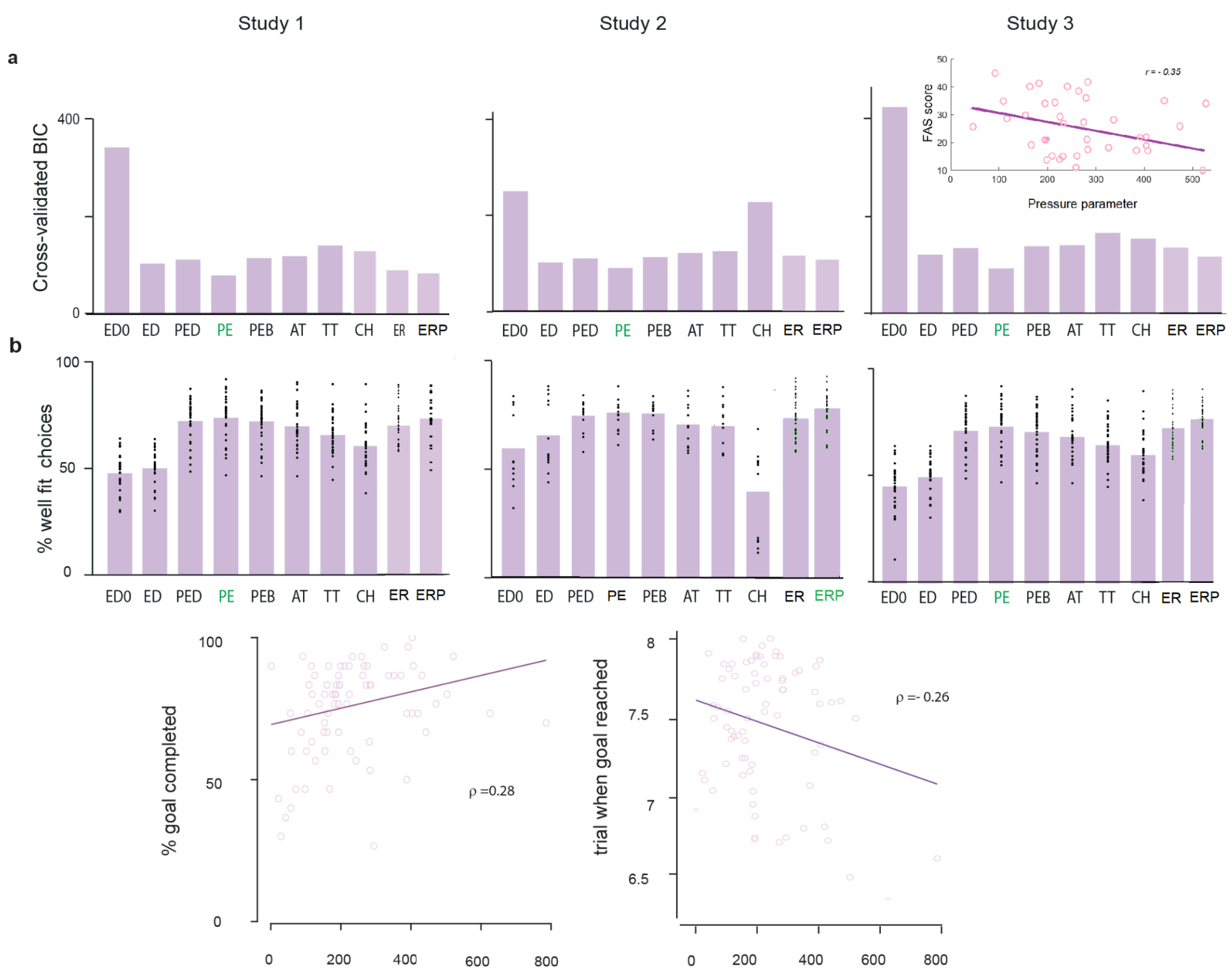

Study 1
Study 2
Study 3
a
Cross-validated BIC
400
0
ED0 ED PED PE PEB AT TT CH ER ERP
FAS score
r = - 0.35
Pressure parameter
b
% well fit choices
100
50
0
ED0 ED PED PE PEB AT TT CH ER ERP
% goal completed
ρ =0.28
trial when goal reached
ρ =- 0.26
0
200
400
600
800

- The dissociation between rostral vs. dorsal ACC for pressure vs. no pressure trials is compelling. I wonder to what extent the "effort no pressure" could be re-considered as a "bias towards pursuing high reward". That is, the contextual difference of having pressure to complete a goal might require critical evaluation of the choices, whereas without the pressure the individual may be deciding which action would maximize reward (consistent with past work in this domain). This does not refute the current finding or interpretation, but I think it could be helpful to contextualize this finding in light of the broader extant literature and should be clarified in the Discussion.

We thank the reviewer for this observation and they raise an interesting point. There are several possible interpretations of this result. Indeed when pressure is zero people could either be simply responding to higher reward, picking that option without deliberation. Although notably, people still have to exert the effort of that option to obtain that reward, and thus the value of that reward might still be discounted by the effort required. As such, it is not clear-cut that participants are not evaluating the option. Indeed ACC signals the value of rewards discounted by effort even in paradigms where there is no choice (Croxson et al., 2009, *Journal of Neuroscience*). In line with the reviewers other comment below, we did not want to further lengthen our discussion of ACC, as although the reviewers point is interesting the discussion section is aiming to address multiple rounds of reviews comments. In our reformulated and more succinct discussion we do however emphasise that activity in the ACC is likely to perform a spectrum of computations which include representations of effort, reward, value and pressure. Line 635-643 now read:

Notably, we found a spectrum of computations performed across multiple sub-regions of the MCC and ACC with the chosen effort signalled when it was being processed as a cost or when it was being sought. Another distinct sub-region signalled subjective value according to our computational model. This would suggest that there might be different mechanisms that guide effort-based choices when aiming to reach a goal, compared to when there is no goal, that ACC ***might include representations of effort, reward, value and pressure in different subregions at different times*** *and argues against the idea that our results could be explained by one alternative factor such as choice difficulty.*

- It is not clear that Choice Value analyses (high effort vs. low effort option) does not actually reflect the subjective evaluation of effort accurately, as effort offer is confounded with reward value (high reward was also yoked with low effort, low reward was always yoked with high effort). Are these analyses controlling for reward amount? Perhaps I am misunderstanding this figure, but it is not clear whether the analyses in Figure 4d reflect differences in reward value or effort value – if effort is higher than would we not anticipate activation to correspond to high effort / low value options? Clarification of these analyses and the corresponding figure is important to substantiate the author's claims about neural activation to choice value. It is not clear that the evidence in the current form how these choices under pressure relate to subjective value of effort-based choice.

We thank the reviewer for their point which allows us to clarify that our analyses are indeed controlling for the reward amount as the "value of an option" is proportional to the reward (equation 5). In the task, the effort of both offers varied independently from the reward of

both offers, and as such activity related to one variable (e.g. effort) would not necessarily be driven the value of the other (e.g. reward). In the computational models the subjective value of an option depends on both the reward offered and effort required to obtain it. In the figure we plot the difference in subjective value between the options.

In figure 5d (which we believe was the figure the reviewer was referring to) the label reading “Value (high effort vs low effort option) was perhaps misleading and suggestive that we ignored the reward to compute value. This was not the case. We therefore changed the label into “value difference (signed)” and now emphasise in the caption that is the value difference between the options according to our equation.

In the methods section we also clarify:
The levels of effort and reward on offer for both options were independently varied on each trial.

Minor comments
- Line 199, this sentence is difficult to parse: “Secondly, as outlined above we showed that people sought the higher effort option more often the more it was higher in effort and also were more likely to choose the lower effort option when this lower effort was closer to the higher level.” What do the authors mean by that people choosing the lower option level when lower effort was closer to the higher level? I cannot make sense of this sentence – it would be helpful to clarify the various decision option labels.

We apologise for the lack of clarity. It is important to note that both effort levels of both options were variable across trials and so the effort level of the lower effort level could be higher on some trials than others.

What we found was that people were more likely to choose the lower effort option in trials when it was higher in effort, thus they were not preferring the lowest effort level option. This mirrors the fact that in studies 1 and 2 they are choosing the higher effort option more often when it was higher in effort. That is people are preferring the higher effort levels of both options.

We have now modified this sentence as follows:

Secondly, as outlined above we showed that people sought the higher effort option more often the more it was higher in effort. They were also more likely to choose the lower effort option in trials when this was at its higher effort level

- Line 469 – “we replicated in three experiments the finding that people seek higher effort behaviors, that are associated with lower immediate rewards” -- This is not accurate, this should be revised to reflect the finding about pressure and the claims about high effort effects only should be tempered considering the evidence presented in the manuscript.

We reformulated to reflect the finding about pressure. The sentence now reads:

We replicated in three experiments the finding that pressure drive people to seek higher effort behaviours, that are associated with lower immediate rewards

- There are numerous typos in the Discussion, please check for for grammatical errors.

We fixed the typos, thank you for highlighting this issue.

- The discussion section overall is quite lengthy and longwinded. This could be streamlined to focus on the key points of the paper, as well as contextualize the authors' findings (which are exciting!) and how they update our understanding of these neural and computational mechanisms of effort-based decision making instead of primarily pontificating on the paradoxical nature of effort or other factors beyond the scope of the study (i.e., procrastination). WRT the past theories on ACC/MCC function, rather than listing all the past theories, it may be useful to clarify to what extent the current findings are consistent or are inconsistent with past theory/data, as it is well known that this region is heterogeneous and interpretations idiosyncratic based on the researcher and experiment. Trimming this section would help the reader better understanding the novelty of these findings.

We thank the reviewer for their point. We appreciate our discussion is rather long. This is in large part due to the fact that the manuscript has been amended to incorporate comments from previous reviewers in multiple rounds. Doing justice to their comments alongside two new reviewers is important. Nonetheless we have now trimmed several sections, including the one about past theories on ACC/MCC functions and removed the sections on procrastination.

- This paper mentions ultra-high-field MRI as a feature of this study – but did not discuss how this would benefit over regular MRI. Is the main advantage that there is better tSNR in the subcortical regions (e.g., putamen)? Would be helpful to mention how this was an advantage in this study.

We thank the reviewer for this point. Indeed ultra-high field MRI affords for higher SNR across the brain, giving more statistical power in samples of this size and therefore increasing potential for looking at subcortical regions as well. We now comment on this at lines 629-631 where we wrote:

Incidentally, we note that the higher signal to noise ratio afforded by ultra-high field MRI provided the resolution to better distinguish subcortical regions across the putamen.

In addendum to my review, I have been requested to comment on the response to the author's rebuttal to R2 + R3 critiques.

Overall, I find that the authors are responsive to these reviewers' comments. However, I do have some minor concerns, detailed below.

We thank the reviewer for positively assessing our earlier rebuttal and for the additional points that allowed us to further clarify our narrative.

The authors state “Without a longer-term goal and pressure to reach this by a deadline, people would always choose more reward and less effort”, which is may not be entirely accurate as this hinges on the normative assumption that on a given decision the receipt of monetary reward is endogenously more valuable than exerting effort. For instance, this would not be true in the case where people entirely discount the value of money (or whatever reward) or may find intrinsic value in selecting the high-effort option.

We thank the reviewer for their point with which we agree. We were responding to a point of the earlier reviewer to emphasise the change from the classic effort-avoiding behaviour but we recognise that there are also many typical behaviours where people choose effort even in the absence of pressure to reach a goal and we should have written “often”, not “always”. Consistently we are keen to avoid the normative assumption that rewards are “always” more valuable than effort in the manuscript and in the revised discussion where we refer to the paradoxical nature of effort paradox we state that (lines 33-36).

Anecdotally people undertake highly effortful behaviours associated with little immediate extrinsic reward with regularity, such as training for a marathon or climbing a mountain, or pursuing long term pro-social goals, suggesting effort is not always avoided.

The authors make the following claim: "Typically making this progress requires effort, both in our task and in everyday life, so people choose higher effort when under high deadline pressure to make as much progress as possible and thus ensure they reduce the pressure and complete goals in time."

I do have a concern about this claim. This is likely true in the context of this study since the amount of effort required is not disproportionately larger than the amount of expected reward/benefit for exerting that effort. For instance, if an individual is completely incapable of exerting the force required, then they would never make that choice. I think it would help to include some language limiting the scope of when higher effort is selected under deadline pressure, stating the caveat that these effects likely are driven when effort is proportional (or comparable to) expected reward. While investigating the boundary conditions of when effort is no longer worthwhile or beneficial in the progress-towards-goal context is outside of the context of the current study, stating these experimental limitations is important for the reader to understand how generalizable these effects are (or not) to everyday life.

We thank the reviewer for these sensible points with which we agree. To highlight them in the text we added a paragraph on this limitation of our work highlighting it as a future direction, in lines 665-673:

More generally, our studies are limited to goals, which are relatively easy to reach, and effort levels, which are relatively easy to exert. It will be important to explore the boundaries of this effort-seeking behaviour in pursuit of goals in everyday life, understanding under which conditions people deem goals unattainable and effort levels unachievable and whether these judgements are driven by considerations around the proportionality of the effort in relation to the expected reward of the goal.

Reviewer #4 (Remarks on code availability):

I was not able to review the code as the repository was set to private required to request access to review. I did not do this because it would have unblinded my review. It would be helpful to change the settings of the OSF to allow for code review by a general audience, to evaluate the reproducibility of the code generated by this project.

We apologise to the reviewer, we have now changed the settings on OSF.

Reviewer #5 (Remarks to the Author):

For context, I have been asked to evaluate whether this revision has addressed the previous reviewers' outstanding concerns.

With regards to the comments to the framing of the results—in particular, the "effort adds value" interpretation—I believe the language used in the revision now appropriately interprets these results as: deadline pressure can induce effort expenditure (even for small reward amounts).

We thank the reviewer for positively assessing our revision which allowed us to improve the narrative.

However, I am not convinced that the results of Experiment 4 (where no effort was required to advance goal progress) demonstrates anything meaningful over and above the existing studies. That is, I simply cannot see how the result that subjects are slightly less likely to choose options which confer greater progress (fig 9E) when there is no effort required as support for the idea that effort imbues goal progress with value. First, the key comparison depicted in Fig 9E is made between experiments, rather than conditions within an experiment, in the absence of random assignment (which, in my view, precludes the use of inferential statistics like t-tests across experiments). The magnitude of this difference is small, and I would expect that, across identical replications of this choice task, this overall preference would move slightly around between experiments simply owing to noise. So what conclusion can be drawn on the basis of comparing these two different proportions that were obtained at different times (e.g. study 3 and study 4) with different samples and different designs? According to psychological research methods orthodoxy: very little.

We thank the reviewer for raising this point with which we largely agree. This experiment was explicitly requested by a previous reviewer and editorially in a previous round. We feel that, while it adds some value (and is thus worth including) to the interpretation to say that pressure influences choices of how to complete goals in the absence of effort, it does not change how we should interpret the main results of our other studies upon which our main conclusions are drawn. To this end, it was not our intention to over emphasise this study or draw too strong conclusions based on comparisons between the pressure effect in the presence of or absence of effort across the studies. We have however removed references to the difference in the discussion, and toned down the caption and labels of Suppl. Fig. 9 where we now simply state that "Progress is chosen as or more often when associated to effort than when not".

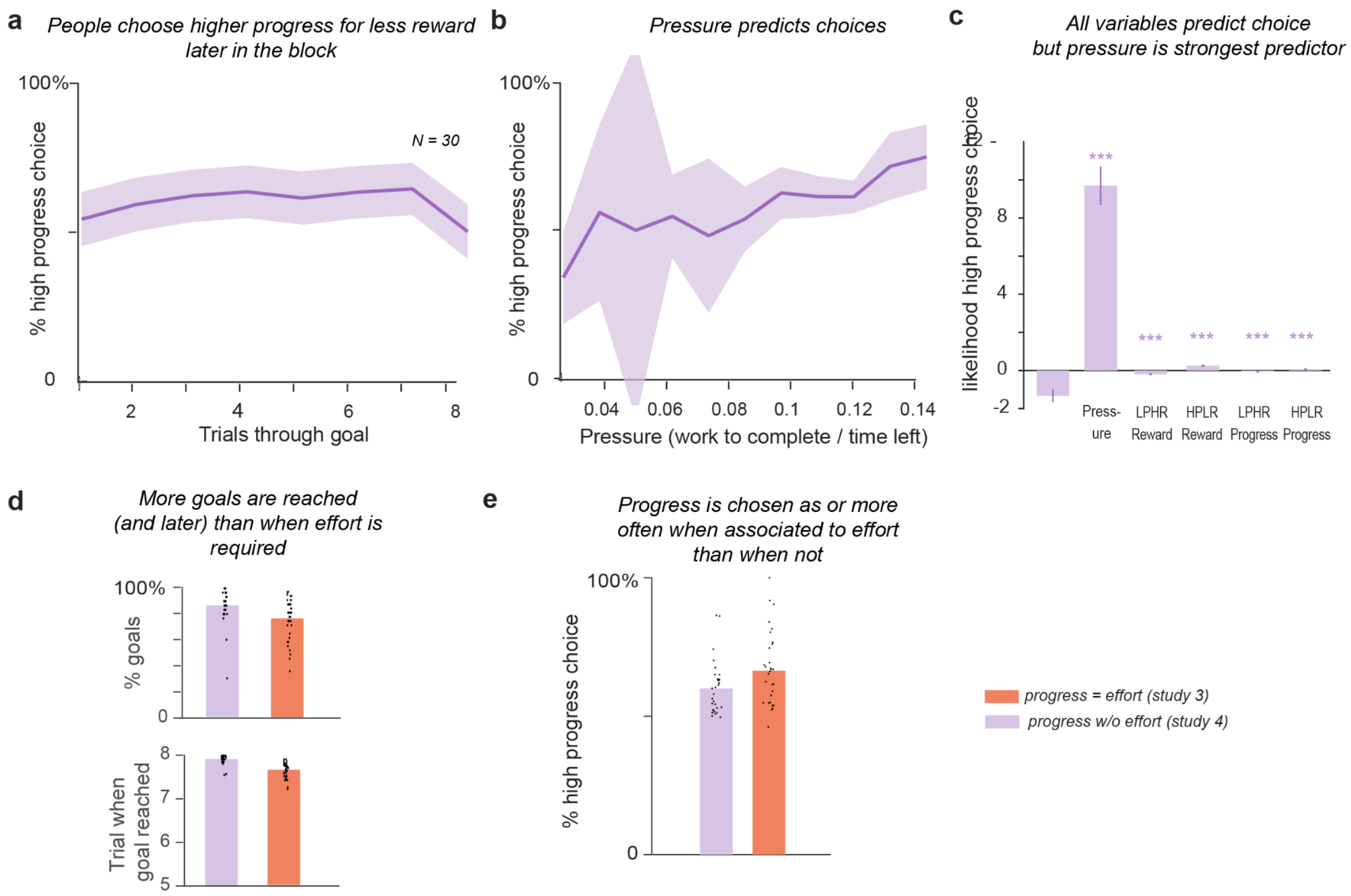

Finally, given that this study specifically examines physical effort I wondered why the authors used such a broad "effort" framing instead of a more specific framing about the type of effort considered here (physical). This framing used throughout the paper struck me as rather underconstrained because it gives the impression that the findings concerning effort perception or fatigue pertain to effort in general—but in reality these findings only speak to physical effort.

We thank the reviewer for raising this point. It is fair to say that our conclusions are constrained by the physical nature of the effort deployed, however, we would note that we did use more than one manipulation of effort (button clicks vs grip force) to increase the generalisability. In addition, there are reasons to believe the findings may translate to cognitive effort as well. Firstly, there is significant overlap in the regions we identified in this study (e.g. regions of medial frontal cortex), with regions that are involved in processing the value of exerting both cognitive and physical effort. Indeed in one study which matched cognitive and physical effort tasks as much as possible, they found largely overlapping regions of frontal cortex signalling the value of rewards discounted by effort, with only the amygdala showing a dissociation (Chong et al., 2017). Secondly, conceptually, there is little reason why pressure would act fundamentally differently for a different type of effort. People still need to reach goals through cognitive effort, in the same way as they do physical effort, and more progress is made on tasks through more cognitive effort. For these reasons we feel that the current framing reflects the fact that features of motivates are similar between different types of effort. However, we agree that this needs to be tested in future research to make strong claims. Thus, in the revised manuscript we make this much clearer that future studies will need to assess the role of pressure in motivating cognitive effort in lines 660-664

“Furthermore, in our studies we only tested the impact of goal pressure on physical effort. While it is likely that the motivational element of pressure would extend to cognitive effort, future studies will need to assess whether and to what extent pressure induces effort seeking behaviour in the cognitive domain as well. ”

Moreover, we have highlighted the physical nature of the effort in the abstract and in a few places in the discussion to ensure that we do not over interpret our findings.

REVIEWERS' COMMENTS

Reviewer #4 (Remarks to the Author):

The authors did a fantastic job of thoroughly addressing all my comments, which I greatly appreciate (and acknowledge is a challenge with multiple rounds of review). I am happy to recommend that the manuscript for acceptance. Congrats on a comprehensive paper!

P.S. There is a minor typo on page 8 (not sure what supplementary figure X is referring to).

We corrected the typo. We thank very much the reviewer for their comments that helped us improve our manuscript and for our appreciation of our work.

Reviewer #5 (Remarks to the Author):

I am by and large satisfied with the authors' revisions made in response to my concerns and I am happy to recommend publication.

We thank the reviewer for their comments that helped us improve our manuscript and for our appreciation of our work.
